# Interacting effects of habitat structure and seeding with oysters on the intertidal biodiversity of seawalls

**DOI:** 10.1371/journal.pone.0230807

**Published:** 2020-07-16

**Authors:** Elisabeth Marijke Anne Strain, Vivian Ruth Cumbo, Rebecca Louise Morris, Peter David Steinberg, Melanie Jane Bishop

**Affiliations:** 1 Sydney Institute of Marine Science, Mosman, New South Wales, Australia; 2 Centre for Marine Science and Innovation, School of Biological, Earth and Environmental Sciences University of New South Wales, Sydney, New South Wales, Australia; 3 Department of Biological Sciences, Macquarie University, Macquarie Park, New South Wales, Australia; 4 Centre for Research on Ecological Impacts of Coastal Cities, School of Life and Environmental Sciences, The University of Sydney, Sydney, New South Wales, Australia; Maurice Lamontagne Institute, CANADA

## Abstract

The construction of artificial structures, such as seawalls, is increasing globally, resulting in loss of habitat complexity and native species biodiversity. There is increasing interest in mitigating this biodiversity loss by adding topographic habitat to these structures, and/or seeding them with habitat-forming species. Settlement tile experiments, comparing colonisation of species to more and less complex habitats, have been used to inform eco-engineering interventions prior to their large-scale implementation. Most studies have focused on applying one type of intervention (either adding habitat structure or seeding with native organisms), so it is unclear whether there are greater benefits to biodiversity when multiple interventions are combined. Using a fully orthogonal experiment, we assessed the independent and interactive effects of habitat structure (flat vs. crevice/ridges) and seeding with native oysters (unseeded vs. seeded) on the biodiversity of four different functional groups (sessile and mobile taxa, cryptobenthic and pelagic fishes). Concrete tiles (flat unseeded, flat seeded, complex unseeded and complex seeded) were deployed at two sites in Sydney Harbour and monitored over 12 months, for the survival and colonisation of oysters and the species density and abundances of the four functional groups. The survival of seeded oysters was greater on the complex than flat tiles, at one of the two sites, due to the protective role of crevices. Despite this, after 12 months, the species density of sessile invertebrates and the percentage cover of seeded and colonising oysters did not differ between complex and seeded tiles each of which supported more of these variables than the flat unseeded tiles. In contrast, the species density of mobile invertebrates and cryptobenthic fishes and the MaxN of pelagic fishes, at 1 month, were only positively influenced by seeding with oysters, which provided food as well as habitat. Within the complex seeded and unseeded tiles, there was a greater species density of sessile taxa, survival and percentage cover of oysters in the crevices, which were more humid and darker at month 12, had lower high temperature extremes at months 1 and 12, than on the ridges or flat tiles. Our results suggest that eco-engineering projects which seek to maximise the biodiversity of multiple functional groups on seawalls, should apply a variety of different microhabitats and habitat-forming species, to alter the environmental conditions available to organisms.

## Introduction

Habitat complexity, the physical structure of an environment, is one of the key drivers of species biodiversity [1–3]. At small scales, habitat complexity is frequently correlated with an increased species diversity, species density and abundance of organisms [4, 5]. Complex habitats can promote species coexistence by providing a wider range of niches that reduce niche overlap [6, 7] and by increasing the area available for organisms to occupy [1]. Additionally, habitat complexity may influence species diversity, species density and abundances by weakening predator-prey interactions [8], by dampening the effects of disturbance [9], by determining the range of organismal body sizes that can be supported by an environment [4, 10], and by intercepting and retaining organic matter [11].

The critical role of habitat complexity in determining species diversity, species density and abundance is of concern, given the ongoing habitat loss and homogenisation [2]. In urban marine environments, natural habitats are being replaced by artificial structures (e.g. seawalls, groynes, breakwaters, wharves) with reduced complexity [12–14]. The smooth, relatively homogenous, surface of these structures typically support a reduced species diversity and species density as compared to the complex natural habitats they replace [6, 15]. There is increasing interest in how complexity might be incorporated into the design of urban marine structures so as to enhance their ecological value [16–19]. Both the addition of topographic habitat structure (e.g. grooves, pits, ledges, water retaining structures; hereafter referred to as habitat structure) and habitat-forming species (e.g., corals, bivalves and seaweeds) have been proposed as mechanisms by which the biodiversity of these structures might be enhanced [19].

The type of complexity added and whether it is structural or organismal (i.e. habitat-forming species) may influence its impact on the species density and abundances of colonising and associated organisms. As animals of different body size utilise space differently, certain scales of habitat structure may disproportionately benefit particular species that fit within their interstices [10, 20], providing them with protection from predators and/or abiotic stressors. Additionally, organisms vary in their niche requirements, so depending on the type of microhabitats that the complexity creates, different taxa may be promoted [19]. For example, on intertidal marine hard substrates, whereas algae and sessile invertebrates are each limited in abundance by the availability of space for attachment and grazing, algae have the additional requirement of a moist microhabitat, that prevents desiccation, and adequate light for photosynthesis. The addition of habitat structure that promotes water retention in well-lit environments may disproportionately enhance algae [21–23]. Sessile invertebrates in contrast, could benefit from microhabitats that provide protection from predators, but that are shaded, so that algal competitors cannot survive [24–26].

Whether complexity is provided by structural features or habitat-forming species may influence the longevity of effects on the species density and/or abundances of colonising organisms. In contrast to abiotic habitat structure, the complexity provided by habitat-forming species may be more dynamic, varying as a function of habitat-former growth, and mortality [27]. Nevertheless, even where habitat-forming species are short-lived, they may have important ongoing effects by influencing the initial trajectory of species colonisation [28]. For example, habitat-forming species that are dominant space occupants may inhibit some species from colonising by pre-empting settlement space [29, 30]. Alternatively, in ameliorating particular abiotic and biotic stressors, they may facilitate the survival of colonising species (e.g. sessile or mobile taxa or cryptobenthic fishes) in otherwise unfavourable environments, and enable them to recruit to size classes or developmental stages at which they can then persist, even in the event of the demise of the habitat-forming species [31, 32]

Additionally, where habitat-forming species provide the structure, their influence on biological communities may not only reflect their structural attributes but also their biological functions [33, 34]. For example, the facilitation of colonising organisms by habitat-forming bivalves is not only a result of their structure, but also their filtration modifying patterns of settlement [35] and producing pseudofaeces, which in turn can provide food and habitat to infaunal taxa [36]. Additionally, bivalves may serve as prey items for some fish and invertebrate species, such that their influence on biodiversity is also trophic [37].

Small-scale experiments, investigating how different types of complexity enhance biodiversity, can be informative in designing eco-engineering interventions that enhance the biodiversity of artificial structures at larger spatial scales, and across a range of environmental settings. These experiments may manipulate complexity by modifying abiotic habitat structure (e.g. by adding pits, crevices or towers), or through the transplant of habitat forming species, such as bivalves, corals and seaweeds [19]. The majority of research has assessed effects of abiotic habitat structure and habitat-forming species on biodiversity independently of one another, but there may be benefits of adding the two together, particularly if the two forms of complexity interact synergistically [7, 38]. The interactive effects of abiotic habitat structure and habitat-forming species are largely untested on seawalls, particularly for cryptobenthic and pelagic fishes that may use complexity for shelter or foraging [39]. Positive feedbacks between the two forms of complexity may occur where habitat structure protects habitat-forming species from predators and/or other environmental stressors (i.e. high maximum or variation in temperatures, desiccation and wave exposure) allowing them to persist through time and support dense and diverse assemblages [40, 41]. However, in other instances there may be redundancy in their combined effects [39] or they may act independently [42].

Recent studies of tiles attached to seawalls demonstrated that, cryptobenthic fishes interacted more with structurally complex than flat tiles [39], while pelagic fishes interacted more with tiles seeded with native bivalves and/or algae relative to unseeded tiles [7, 39]. In general, the effects of these small-scale manipulations were greater for cryptobenthic than pelagic fishes [39]. This is because cryptobenthic fishes spend more time on complex habitats, as they are better at avoiding predators in crevices than on flat surfaces [39]. Seeding may, however, also influence patterns of habitat utilisation by pelagic fishes if the seeded organism represents a prey item for them or enhances the availability of prey items relative to naturally fouled tiles [39], or adjacent habitats [27]. These results suggest that adding habitat structure and seeding with native bivalve on tiles can influence the behaviour of both cryptobenthic and pelagic fishes [27, 39] however the effects on species density and MaxN through time remains unclear.

Here we conduct a small-scale intertidal tile experiment in an urban context to assess how manipulation of habitat structure, through the addition of cervices and ridges and seeding with oysters to tiles, interact to influence the species density and abundances of sessile taxa, mobile invertebrates, and cryptobenthic and pelagic fishes. The tiles are affixed to vertical seawalls to match the orientation of many artificial structures in the marine environment. We hypothesise that in contrast to the temporally persistent complexity of crevices and ridges, the habitat provided by transplanted oysters will be temporally dynamic due to processes such as mortality and growth. Nevertheless, even in the event of considerable mortality of the oysters, we predict that initially seeded tiles will support distinct assemblages to those that are unseeded, due to effects on species assembly that occur early in the colonisation process. Additionally, we expect that the presence of crevices will enhance oyster survivorship by providing protection from environmental stressors (i.e. extreme temperatures and desiccation) and reducing algal overgrowth through shading, such that there are non-additive effects of providing the two forms of complexity.

Despite the overall prediction of synergistic effects of crevices/ridges and seeding with oysters on the species density and abundances, we expect that effects of the two factors will vary among the four functional groups of organisms. We expect that sessile taxa, mobile invertebrates and small cryptobenthic fishes (defined here as those associated with the substrate) will respond directly to habitat structure and any amelioration of extreme temperatures, desiccation stress and predation it provides, so will display positive non-additive responses to abiotic habitat structure and oyster seeding. Finally, we predict that pelagic fishes (defined here as those in the water adjacent to tiles) that may utilise oysters as a food resource will spend more time at tiles seeded than unseeded with oysters, resulting in greater species density and abundances associated with seeded tiles.

## Methods

### Permit

Permission to work on the seawalls was obtained directly through the North Sydney and Leichhardt councils and permit P03/0029-4.1.

### Study sites

The experiment was undertaken on sandstone vertical seawalls, which were located at two sites (Morts Bay Park, Balmain, Site 1: -33.854, 151.185, Sawmillers Reserve, Waverton, Site 2: -33.845, 151.200 GPS), within Sydney Harbour, New South Wales, Australia [7]. The sites (1 km apart) were each situated approximately 7 km from the mouth of the harbour, where the semi-diurnal tides have a mean amplitude of 1.62 m. The experiment was conducted in the mid-intertidal zone (i.e. MLWS + 0.7–0.9 m) on each of these seawalls, where low density populations (relative to nearby natural rocky shores) of the native bivalve Sydney rock oyster, *Saccostrea glomerata*, naturally occurred [43].

### Experimental design

**Fig 1:**
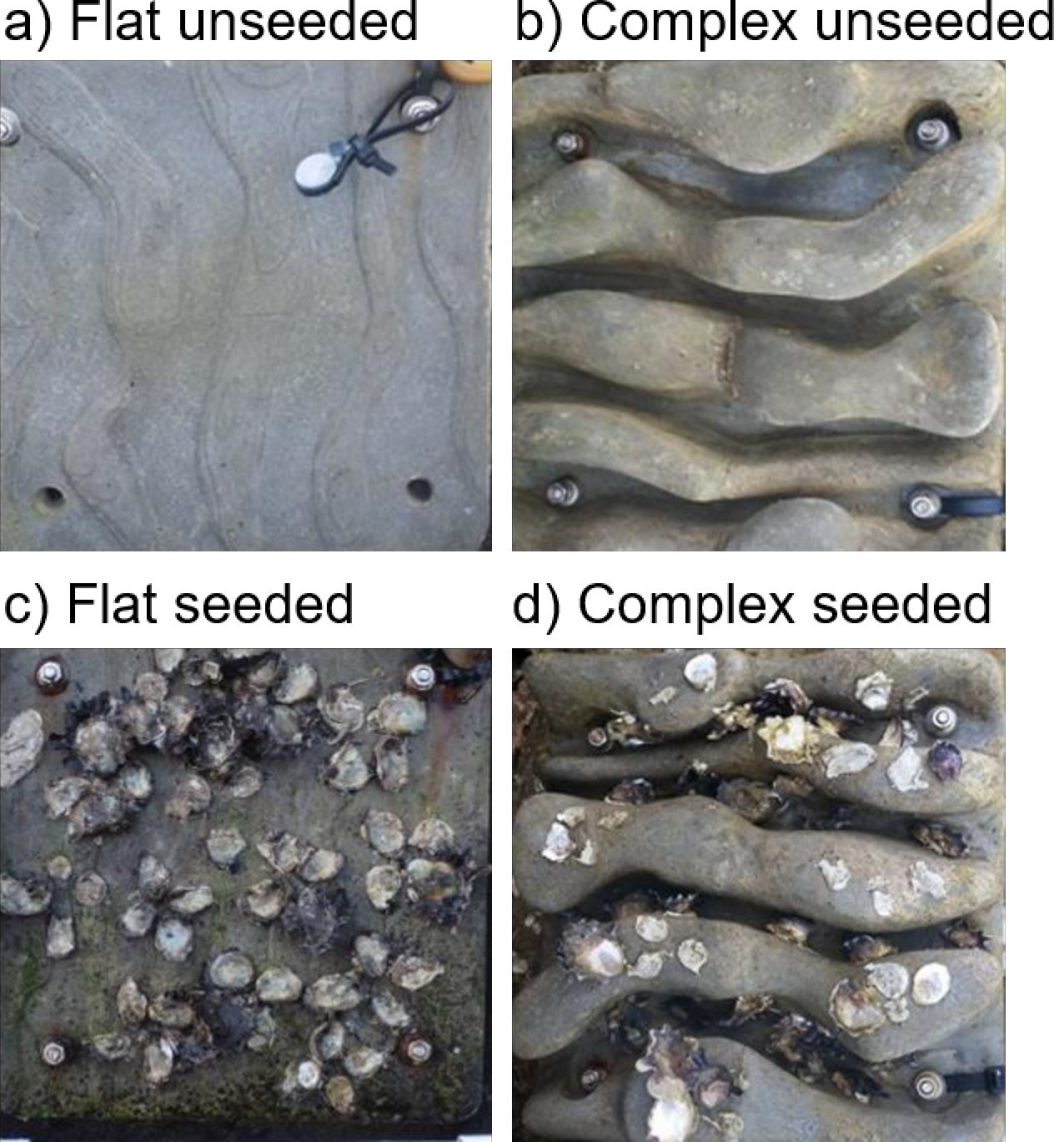
The four experimental treatments: a) flat unseeded tiles; b) complex unseeded tiles; c) flat seeded tiles; d) complex seeded tiles. Complex tiles had ridges and crevices; seeding was with the Sydney rock oyster, *Saccostrea glomerata*.

We manipulated habitat structure on seawalls using 0.25 × 0.25 m concrete tiles that were either flat (surface area = 0.0625 m^2^) or complex (surface area = 0.136 m^2^) with five 5 cm high ridges, each separated by 1.5 to 5 cm wide crevices (Fig 1). The tiles were produced from moulds designed by Reef Design Lab (Melbourne, Australia) using a marine concrete mix commonly used for seawall construction. The flexible moulds allowed for manufacture of complex geometry with undercuts and surface texture. We transplanted *Saccostrea glomerata*, a habitat-forming oyster, onto a sub-set of tiles of each treatment (resulting in ‘seeded’ and ‘unseeded’ treatments), in a fully orthogonal design (Fig 1). In the Austral spring (November) of 2015, we deployed five tiles of each of the four treatments in random order, approximately 1 m apart, at each of the two sites, to avoid any spatial auto-correlation in the mobile invertebrate observations (26).

For the seeded treatments, 52 juvenile *S*. *glomerata* (mean ± SE shell height: 23.7 ± 3.9 mm) were attached in clumps of 4–5 individuals using non-toxic epoxy glue (RMF—Vivacity Marine Ltd Pty). On seeded flat tiles, the clumps of *S*. *glomerata* were placed haphazardly and on the seeded complex tiles, half of the oysters (i.e. 26 individuals) were attached in clumps to the crest of ridges and the other half (26) were attached in clumps into the crevices. At each site, the tiles were deployed in a single horizontal row. The complex tiles were positioned so that the crevices and ridges were orientated horizontally.

### Sessile and mobile taxa

#### *In situ* sampling

One, 6 and 12 months after the tiles were deployed, we assessed the number of live seeded *S*. *glomerata*, the percentage cover of oysters and other sessile taxa, and the abundances of mobile taxa on each tile, at low tide during the day. These time points were selected to ensure that sampling was conducted at the beginning, middle and end of the experiment, however month was considered a random factor in the analyses (see below) because we had no *a priori* expectations about the effects of the treatments at these sampling points and our goal was to assess the effects of the treatments over the entire duration of the experiment. The percentage cover of oysters included both live and dead, transplanted and colonising oysters summed together as it was not to distinguish between these categories in the photoquadrats. For each tile, we sampled the percentage cover of *S*. *glomerata* and other sessile taxa with 0.25 × 0.25 m photoquadrats, taken at a distance of 0.5 m from the tiles, using an Olympus Tough style camera (TG-860, mode Automatic mode, 16 MP). Using Coral Point Count software (CPCe) [44], we overlaid 100 randomly generated points on the flat tiles, or 50 points in each of the crevices and ridge crests of the complex tiles. We sampled the same total number of points (i.e. 100, 10 x zoom) on each of the flat and complex tiles to ensure sampling effort was consistent between treatments. The *S*. *glomerata* and other sessile taxa directly under these points (either primary colonisers, directly attached to the substrate, or secondary colonisers, attached to another organism) were summed to calculate the percentage cover per tile (out of 100 points) and for each microhabitat (i.e. crevice or ridge: (number of points/50*100)) on the complex tiles. Simultaneously, *in situ* we counted the number of mobile invertebrate taxa (~ 500μm diameter) on each tile by eye, noting their microhabitat (i.e. crevice or ridge) on the complex tiles, as it was not possible to see these organisms in the photoquadrats.

#### Destructive sampling

After 12 months, the tiles were removed from the seawall, individually bagged and frozen at -5°C until census. For each tile, we recorded the identity and percentage cover (both primary and secondary cover) of the sessile taxa (excluding *S*. *glomerata*) separately from one pre-determined crevice (0.016 m^2^) and ridge (0.013 m^2^) on the complex tiles (out of 50%) and from two areas of similar size on the flat tiles. We then removed all the mobile taxa (> 500 μm) from the same predefined areas on the complex and flat tiles, using tweezers and by rinsing the tile area with seawater over a 500 μm sieve. All sessile and mobile taxa were identified to species using a dissection microscope (Leica M125 x10 zoom) or, where this was not possible, morphospecies. To ensure consistency, the same person identified and enumerated the sessile and mobile taxa (listed in S1 Table), for all samples.

### Fish

At the same time intervals (1, 6 and 12 months) as the sessile and mobile communities were sampled, the fishes associated with the tiles were sampled during day-time high tides using GoPro® cameras [7, 38]. A camera was placed ~0.12 m above each tile and attached to the seawall using stainless steel brackets and a camera housing [7, 38]. The cameras faced downwards, so that the entire tile was in view [7, 38]. The cameras were set to take photos every 2 seconds using the GoPro® setting ‘5 MP, wide’, and recorded images for 3 hours, during the high tide [7, 38]. The tiles were sampled haphazardly, over two days per site, using 10 cameras per day. On each day, tiles at least 5 m apart were sampled to avoid any spatial auto-correlation in the fish observations [7, 38].

Using the video footage and relevant literature, we categorised the fish into two groups ‘cryptobenthic’ (associated with the substrate) or ‘pelagic’ (found in open water; listed in S1 Table). Throughout the study most of the pelagic fishes were adults (98%). Therefore, we did not try to classify fish based on life stage. We quantified the number of species and abundance of fish per frame for each of five 30 min periods of video footage (900 photos). The first 30 min of video footage for each replicate tile were excluded from the data collection to avoid disturbance from the snorkelers. On the complex tiles we also noted whether the cryptobenthic fish only, were situated in a crevice or on a ridge. The maximum number of species and abundance recorded in any one single frame during each 30 min block was averaged across the five periods to give a single number (MaxN [45]) per tile and, for the cryptobenthic fish on the complex tiles, microhabitat (i.e. crevice or ridge). The results presented therefore measure the difference in the average maximum number of fishes per treatment, rather than the average number of fishes (hereafter termed abundance) (37). MaxN is a commonly used measure of fish abundance and ensures that no fish is counted twice within the sample period [45].

### Environmental variables

At five periods (0–1, 2–3, 5–6, 8–9 and 11–12 months) after the tiles were deployed, we took measurements of temperature. At each site, we deployed thirty DS1921G Themochron iButton data loggers (Thermodata Pty. Ltd. Warrnambool, Australia). We placed one iButton haphazardly on each flat tile (unseeded and seeded), and two iButtons (one in the crevice and one on the crest of the ridge) haphazardly on each complex tile (unseeded and seeded), either inside the oyster clumps (for seeded tiles) or outside the oyster clumps (for unseeded tiles). The iButtons were waterproofed with Plastidip rubber coating (Plasti Dip International, Blaine, Minnesota, USA). The iButtons were attached to the tile with a cable tie, which was threaded through a plastic straw, glued to the tile with non-toxic epoxy (RMF—Vivacity Marine Ltd Pty). This enabled iButtons to be interchanged, so as to not exceed memory capacity, throughout the experiment, without damaging the tiles or the colonising organisms. The iButtons were programmed to record temperatures at 20 min intervals, across a one-month period, with 0.5°C accuracy. These measurements were used to calculate the minimum, maximum, range and standard deviation of temperature recorded across each month (16).

One, 6 and 12 months, after the tiles were deployed, we took measurements of humidity with six DS1923 Hygrochron iButtons (Thermodata Pty. Ltd) and readings of light with the external fibre optic sensor from a Diving PAM fluorometer (Walz Pty. Ltd.). As the Hygrochron iButtons could not get wet and the Diving PAM could only be used to make spot measurements, readings of humidity and light were limited to low tide, with tiles sampled in random order with respect to treatment over two consecutive days. For each of humidity and light, we took one haphazardly positioned measurement from each flat tile and two measurements from each complex tile (one in the crevice and one on the crest of the ridge). The Hygrochron iButtons took measurements at 2 min intervals for 16 min, with the 8 measurements per tile averaged to give a single value that was used in analyses.

### Analyses

For each of the four functional groups (i.e. sessile taxa excluding *S*. *glomerata*, mobile invertebrates, cryptobenthic fishes, pelagic fishes), we calculated two univariate metrics: (1) species density (i.e. the number of different species per tile, crevice or ridge), and (2) either cover (for sessile organisms), counts (for mobile invertebrates) or MaxN (for fish). Species density and abundance were calculated separately, at the level of tile and, for complex tiles, also separately for crevices and ridges to assess how the two different microhabitats influenced the biodiversity. The species density was calculated at the final time point, 12 months, via destructive sampling for sessile and mobile organisms or *in situ* sampling for cryptobenthic or pelagic fishes, while the abundance metrics were calculated separately at each time point using *in situ* sampling and at month 12 using destructive sampling (for sessile and mobile taxa).

We used generalised linear mixed models (GLMMs) to test the effects of the initial treatment conditions, adding habitat structure (fixed, 2 levels: flat, complex), seeding with oysters (fixed, 2 levels: unseeded, seeded), site (random, 2 levels) and tile surface area (offset, m^2^), on the species density of each of the four functional groups at 12 months. Analogous GLMMs including these same factors as well as month (random repeated measures, 3 levels) were used to test for the effects of adding habitat structure and seeding with oysters on the percentage cover of *S*. *glomerata* (live and dead, transplants and colonising oysters) as well as the abundance or MaxN, of the sessile and mobile taxa and cryptobenthic fishes and pelagic fishes. The effect of adding habitat structure on the number of live seeded *S*. *glomerata* (transplants only) was assessed using a GLMM that excluded the factor seeding.

To determine the effect of microhabitat identity on each of the same metrics for the sessile and mobile taxa and cryptobenthic fish, a second set of GLMMs were run. For species density data collected at 12 months, these had the factors: microhabitat (fixed 2 levels: crevice, ridge), seeding with oysters (fixed, 2 levels: unseeded, seeded), site (random, 2 levels), area (offset m^2^) and tile (random, microhabitat nested in tile nested in seeding and site, 5 levels). Analyses of cover, abundance and MaxN data also included the factor time (random repeated measure, 3 levels). As no pelagic fishes were recorded on the tiles, we did not test the effects of microhabitat identity on the species density or MaxN of this functional group.

A third set of GLMMS tested for differences in the range of temperature (minimum, maximum and standard deviation), humidity and light levels among microhabitats of both the complex and flat plates (2 fixed levels = crevice or ridge vs. flat) and between the two levels of seeding with native oysters (2 fixed levels = unseeded vs. seeded). These analyses also had the factors site, area and time (random repeated measures, 6 random levels for temperature, 3 levels for humidity and 2 levels for light).

All statistical analyses were undertaken in R 3.5.0 [46]. The generalised linear mixed effects models were conducted using the packages lme4 and figures were produced using ggplot 2 [47]. Likelihood ratio tests comparing models with and without the random effects were used to obtain p-values for the random effects. In the models, species density, percentage cover and counts (i.e. mobile invertebrate abundance and fish MaxN) were modelled using a Poisson distribution with a log link function. The average and standard deviation of temperature, average humidity and light levels were modelled using a Gaussian distribution. All models were checked for over-dispersion and spatial and temporal autocorrelation with plots and the residuals were visually inspected for heteroscedasticity. Where appropriate, post hoc comparisons were undertaken using eemeans to identify sources of treatment effects.

## Results

A total of 80 taxa (excluding *S*. *glomerata*, the seeded species) were identified on tiles during the study (S1 Table). These taxa were classified into four main groups: sessile algae and invertebrates (9 taxa), mobile invertebrates (39 taxa), cryptobenthic fishes (7 taxa) and pelagic fishes (13 taxa) (S1 Table). At 12 months, the number of different species recorded across all tiles of each treatment was: flat unseeded—24 taxa; flat seeded—43 taxa; complex unseeded—34 taxa; and complex seeded—40 taxa.

The most abundant taxa identified during the *in-situ* sampling were for each of the four main groups: the barnacle *Elminius modestus* (sessile algae and invertebrates; cover, mean = 19.650%, SE = +/- 1.947%), the conniwink *Bembicium nanum* (mobile invertebrates; count per tile, mean = 2.308, SE = +/-1.342); the oyster blenny, *Omobranchus anolius* (cryptobenthic fishes; MaxN, mean = 0.329, SE = +/- 0.053) and the yellowfin bream *Acanthopagrus australis* (pelagic fishes; MaxN, mean = 0.428, SE = +/- 0.019). Destructive sampling at month 12 revealed the barnacle *Elminius modestus* (cover, mean = 16.996%, SE = +/- 2.358%) was the most abundant sessile species and the limpet *Patelloida mimula* (count per tile, mean = 5.925, SE = +/- 1.089) was the most abundant mobile invertebrate on the tiles. For each analysis, except for the number of live seeded *S*. *glomerata* and the abundance of mobile taxa, there was no significant difference in the effects of treatments between sites (S2–S4 Tables), allowing sites to be pooled together for the post-hoc tests.

### Effect of habitat structure on the number of live seeded *S*. *glomerata*, and of habitat structure and seeding on the percentage cover of oysters

**Fig 2:**
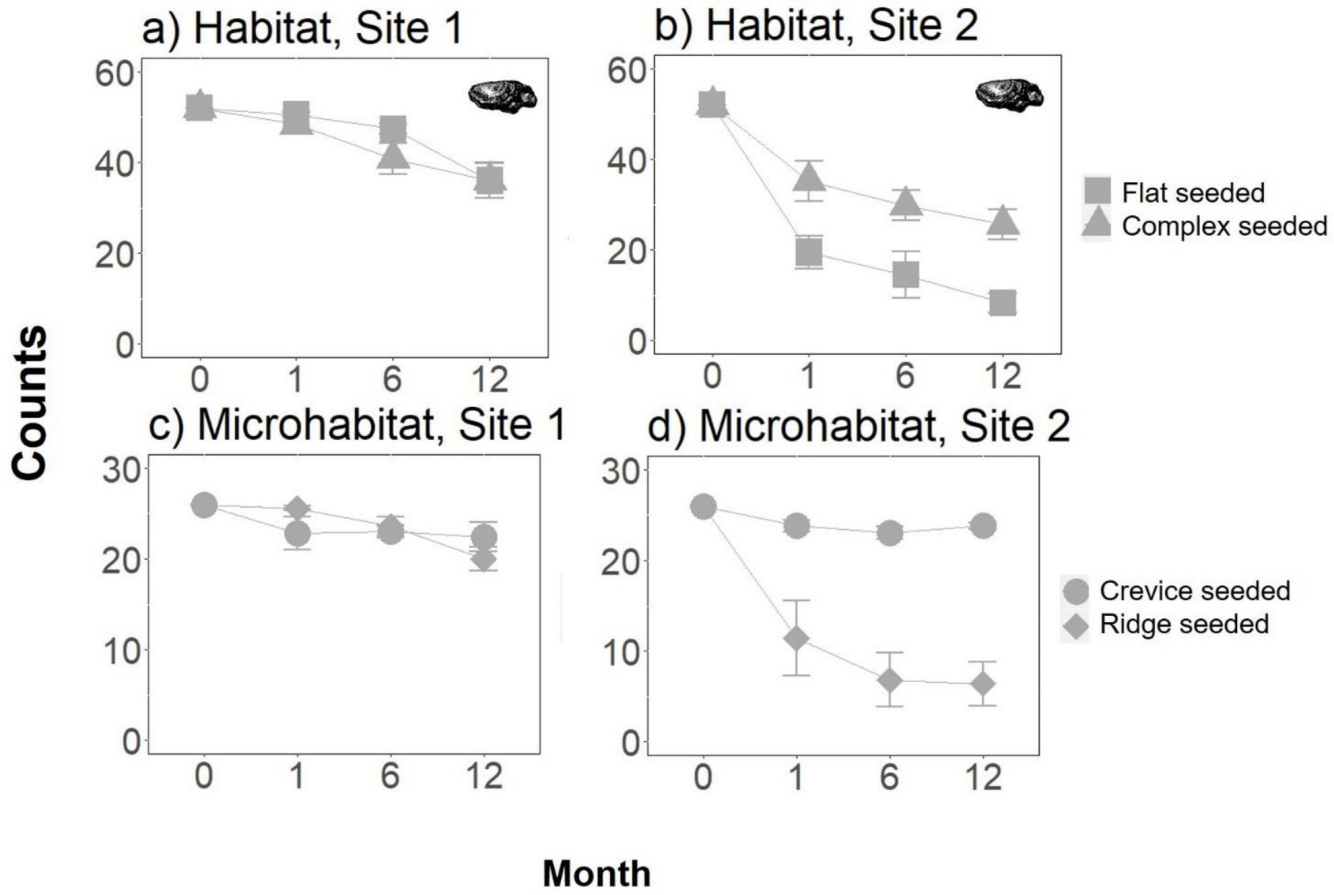
Effect of habitat structure (flat vs. complex tiles; a, b) and microhabitat identity (crevice vs. ridge, on complex tiles; c, d) on the mean (+/-SE) number of live seeded *S*. *glomerata* (transplants only) at each of two sites, 0, 1, 6 and 12 months following tile deployment, (n = 5). Tiles started with 52 oysters.

The effect of habitat structure on the number of live seeded oysters differed between the two study sites (Fig 2A and 2B, S2 Table). At site 1, where survivorship of the oysters was greater, there were no detectable differences in the number of live *S*. *glomerata* between the complex and the flat tiles, at any of the sampling times (Fig 2A, S2 Table). At site 2, where greater mortality was observed, the number of live *S*. *glomerata* was across all sampling times significantly greater on complex than flat tiles (Fig 2B, S2 Table). This difference was driven by the significantly higher number of live seeded *S*. *glomerata* in the crevices than the ridges of the complex tiles at Site 2, but not Site 1 (Fig 2B, S2 Table).

**Fig 3:**
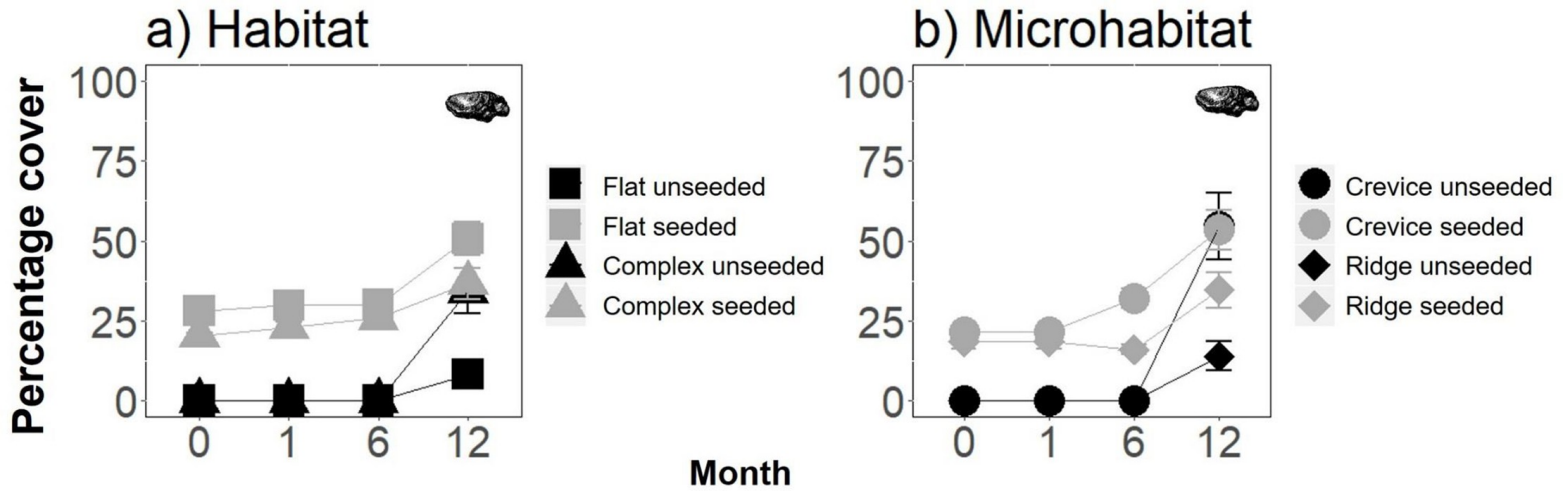
Effect of seeding with oysters (unseeded vs. seeded) and a) habitat structure (flat vs. complex) and b) microhabitat identity (crevice vs. ridge, on complex tiles) on the mean (+/-SE) percentage cover of *S*. *glomerata* (both transplants and recruits) on experimental tiles 0, 1, 6 and 12 months following deployment, (n = 5). Data are pooled across sites.

The effects of adding habitat structure and seeding with oysters on the percentage cover of *S*. *glomerata* (which included both live and dead oysters and seeded as well as colonising individuals) differed through time (Fig 3A, Table 1, S3 Table). At 1 and 6 months, the percentage cover of *S*. *glomerata* was greater on seeded than unseeded tiles, but was unaffected by habitat structure (Fig 3A, S3 Table). By 12 months, the percentage cover of oysters was greater on the seeded complex, unseeded complex and seeded flat tiles (each of which did not significantly differ) than unseeded flat tiles (Fig 3A, S3 Table).

**Table 1:** Summary of the results of general linear models testing for effects of habitat structure on the number of live seeded *S*. *glomerata* and the effects of habitat structure and seeding with native oysters on the percentage cover of *S*. *glomerata*, the species density and abundances (percentage cover, counts or MaxN) of sessile taxa, mobile taxa, cryptobenthic and pelagic fishes. Details of these analyses are given in Appendices S2-S5. ^NA^Not applicable, ^ns^p>0.05, *p < 0.05, **p < 0.01, ***p < 0.001.

Factors	Number of live seeded *S*. *glomerata*	Cover of *S*. *glomerata*	Species density of sessile taxa	Cover of sessile taxa	Species density of mobile taxa	Counts of mobile taxa	Species density of cryptobenthic fishes	MaxN of cryptobenthic fishes	Species density of pelagic fishes	MaxN of pelagic fishes
**Habitat**	^ns^	***	**	^ns^	^ns^	^ns^	^ns^	^ns^	^ns^	^ns^
**Seeding**	^NA^	**	**	^ns^	***	^ns^	*	**	^ns^	*
**Habitat x Seeding**	^NA^	***	*	^ns^	^ns^	***	^ns^	^ns^	^ns^	^ns^
**Site**	***	^ns^	^ns^	^ns^	^ns^	^ns^	^ns^	^ns^	^ns^	^ns^
**Habitat x Site**	*	^ns^	^ns^	^ns^	^ns^	***	^ns^	^ns^	^ns^	^ns^
**Seeding x Site**	^NA^	^ns^	^ns^	^ns^	^ns^	*	^ns^	^ns^	^ns^	^ns^
**Month**	***	***		***		***		***		***
**Habitat x Month**	^ns^	***		^ns^		^ns^		^ns^		^ns^
**Seeding x Month**	^NA^	***		^ns^		***		^ns^		***
**Site x Month**	***	^ns^		^ns^		***		^ns^		^ns^
**Habitat x Site x Month**	*	^ns^		^ns^		^ns^		^ns^		^ns^
**Habitat x Seeding x Site**	^NA^	^ns^	^ns^	^ns^	^ns^	^ns^	^ns^	^ns^	^ns^	^ns^
**Habitat x Seeding x Month**	^NA^	***		^ns^		***		^ns^		^ns^
**Seeding x Site x Month**	^NA^	^ns^		^ns^		*		^ns^		^ns^
**Habitat x Seeding x Site x Month**	^NA^	^ns^		^ns^		***		^ns^		^ns^

Similarly, within the complex tiles, whether seeding, microhabitat type, or an interaction between the two influenced the percentage cover of *S*. *glomerata*, varied through time (Fig 3B, Table 2, S3 Table). Initially, at 1 month, the percentage cover of *S*. *glomerata* was greater in seeded than unseeded microhabitats, irrespective of whether they were a crevice or ridge (Fig 3B, S3 Table). At 6 months, the percentage cover of *S*. *glomerata* was greater in the crevices that were seeded, than the ridges that were seeded, each of which had greater cover than the unseeded microhabitats (which did not significantly differ; Fig 3B, S3 Table). At 12 months, the percentage cover of *S*. *glomerata* was significantly greater in the crevices than in the ridges, irrespective of seeding, with seeded ridges containing greater cover than unseeded ridges (Fig 3B, S3 Table).

**Table 2:** Summary of the results of general linear models testing the effects of microhabitats (crevices/ridges) on the number of live seeded *S*. *glomerata*, and the effects of the microhabitats and seeding with native bivalves on the percentage cover of *S*. *glomerata*, the species density and abundances (percentage cover, counts or MaxN) of sessile taxa, mobile taxa, benthic and pelagic fishes. Details of these analyses are given in supplementary material S4- S5. ^NA^Not applicable, ^ns^p>0.05, *p < 0.05, **p < 0.01, ***p < 0.001.

Factors	Number of live seeded *S*. *glomerata*	Cover of *S*. *glomerata*	Species density of sessile taxa	Cover of sessile taxa	Species density of mobile taxa	Counts of mobile taxa	Species density of cryptobenthic fishes	MaxN of cryptobenthic fishes
**Tile: Microhabitats**	***	***	^ns^	^ns^	***	***	^ns^	^ns^
**Tile: Seeding**	^na^	**	^ns^	^ns^	^ns^	^ns^	*	**
**Tile: Microhabitats × Seeding**	^na^	***	^ns^	^ns^	**	*	^ns^	^ns^
**Tile: Site**	^ns^	^ns^	^ns^	^ns^	^ns^	^ns^	^ns^	^ns^
**Month**	^ns^	^ns^		***		***		^ns^
**Tile: Site x Month**	^ns^	^ns^		^ns^		^ns^		^ns^
**Tile: Microhabitat x Tile: Site**	***	^ns^	^ns^	^ns^	^ns^	^ns^	^ns^	^ns^
**Tile: Seeding x Tile: Site**	^na^	^ns^	^ns^	^ns^	^ns^	^ns^	^ns^	^ns^
**Tile: Microhabitat x Month**	^ns^	^ns^		^ns^		***		^ns^
**Tile: Seeding x Month**	^na^	***		^ns^		^ns^		^ns^
**Tile: Microhabitat x Month x Site**	***	^ns^		^ns^		^ns^		^ns^
**Tile: Seeding x Month x Tile: Site**	^na^	^ns^		^ns^		^ns^		^ns^
**Tile: Microhabitat x Tile: Seeding x Tile: Site**	^na^	^ns^	^ns^	^ns^	^ns^	^ns^	^ns^	^ns^
**Tile: Microhabitat x Tile: Seeding x Month**	^na^	*		^ns^		***		^ns^
**Tile: Microhabitat x Tile: Seeding x Tile: Site x Month**	^na^	^ns^		^ns^		^ns^		^ns^

### Effect of habitat structure and seeding with native oysters on the species density, and abundances of the four functional groups

**Fig 4:**
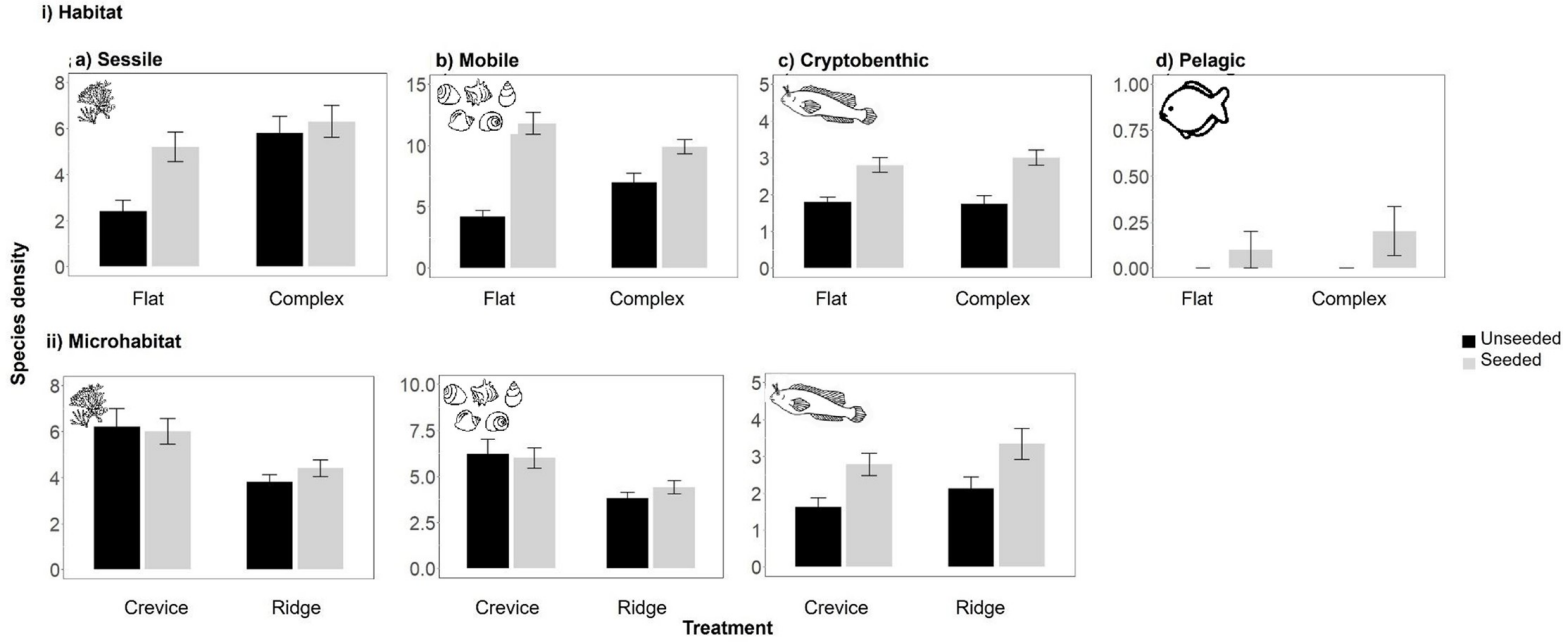
Effect of seeding with oysters (unseeded vs. seeded) and i) habitat structure (flat vs. complex tiles) or ii) microhabitat identity (crevice vs. ridge, on complex tiles) on the mean (+/-SE) species density of a) sessile algae and invertebrates, b) mobile invertebrates, c) benthic fishes and d) pelagic fishes (n = 5). Invertebrates and algae were sampled destructively, and fish were measured *in situ* at 12 months following tile deployment. Data are pooled across sites.

The effects of the treatments on the species density differed among the four functional groups (Fig 4, Table 1, S4 Table). Whereas for sessile taxa, species density responded to the interacting effects of habitat structure and seeding with oysters, for mobile species and cryptobenthic fish, species density was only influenced (positively) by seeding with oysters, and for the pelagic fish there were no detectable effects of habitat structure or seeding with oysters on species density (Fig 4ic–4D, Table 1, S4 Table). The species density of colonising sessile taxa was greater on the flat seeded, complex unseeded and complex seeded tiles (each of which did not significantly differ) than for the flat unseeded tiles (Fig 4ia, S4 Table). In contrast, the species density of mobile invertebrates and cryptobenthic fishes was greater on the seeded flat and complex tiles than the unseeded flat and complex tiles (Fig 4ib–4C, S4 Table).

Within the complex tiles, the effects of microhabitat identity and seeding on species density at 12 months also varied among functional groups (Fig 4ii, Table 2, S5 Table). Whereas for the sessile taxa, species density was greater in crevices than on ridges (Fig 4iia), irrespective of seeding, for mobile invertebrates, species density was greatest in the seeded and unseeded crevices, followed by the seeded ridges and then the unseeded ridges (Fig 4iib) and for cryptobenthic fishes, species density was only influenced (positively) by seeding (Fig 4iic, Table 2, S5 Table).

**Fig 5:**
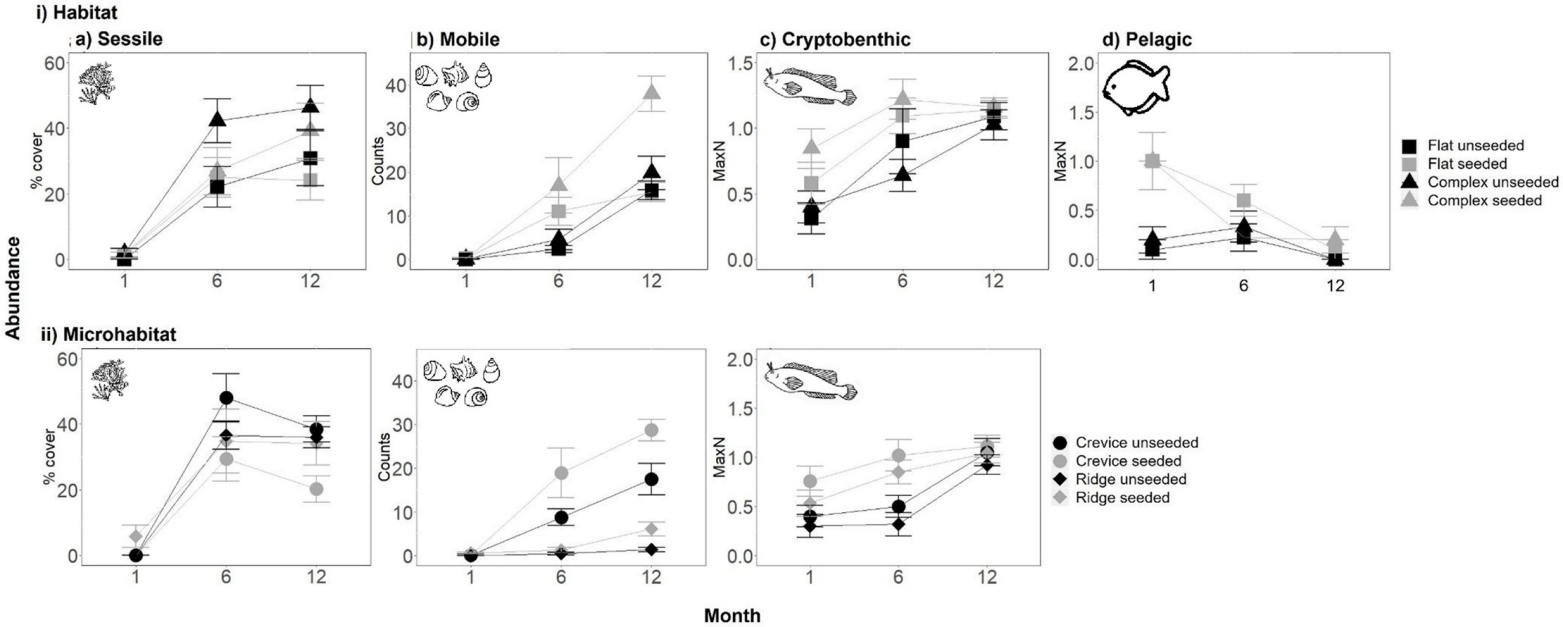
Effect of seeding with oysters (unseeded vs. seeded) and i) habitat structure (flat vs. complex tiles) or ii) microhabitat identity (crevice vs. ridge, on complex tiles) on the mean (+/-SE) a) percent cover of sessile algae and invertebrates, b) count of mobile invertebrates, c) MaxN of benthic fishes and d) MaxN of pelagic fishes sampled *in-situ* 1, 6 and 12 months following tile deployment (n = 5). Data are pooled across sites.

Both the *in situ* and destructive sampling revealed that neither the addition of habitat structure nor seeding with oysters affected the cover of sessile taxa (Fig 5ia, Table 1, S5 and S6 Tables). In contrast, *in situ* sampling revealed that the abundance of mobile invertebrates was significantly influenced by interacting effects of the two treatments, month and site (Fig 5iabc, Table 1, S5 Table). At 1 month, and for both sites, there was no effect of adding habitat structure or seeding with oysters on the abundance of mobile invertebrates (Fig 5ib, Table 1, S5 Table). At 6 months, and for both sites, there were significantly higher numbers of mobile invertebrates on the unseeded and seeded complex tiles than the unseeded and seeded flat tiles (Fig 5ib, Table 1, S5 Table). At 12 months, and for both sites, there were significantly higher numbers of mobile invertebrates on seeded complex tiles than on any other treatment (Fig 5ib, S5 Table). The destructive sampling of mobile invertebrates after 12 months, however, revealed a greater abundance on the complex or flat seeded tiles (which did not significantly differ), than either the complex unseeded tiles or the flat unseeded tiles, with the latter treatment recording the fewest mobile invertebrates (S6 Table). The MaxN of the cryptobenthic fish was much higher adjacent to the seeded than the unseeded tiles through time (Fig 5ic, S5 Table) while the MaxN of pelagic fishes was greater for seeded than unseeded tiles at 1 month, although this effect subsequently disappeared at 6 and 12 months (Fig 5D, S5 Table).

Within the complex tiles, the percentage cover of sessile taxa was unaffected by microhabitat type or seeding with native oysters, irrespective of whether sampling was *in situ* or destructive (Fig 5iia, Table 2, S5 and S6 Tables). In contrast, the abundance of mobile invertebrates was significantly influenced by the interacting effects of these two treatments, the effects of which varied through time (Fig 5iib, Table 2, S5 and S6 Tables). At 1 month, there was no effect of microhabitat type or seeding with oysters on the abundance of mobile invertebrates. At 6 months, there was a greater abundance of mobile invertebrates in the crevices than the ridges and by 12 months, both the *in-situ* and the destructive sampling showed the number of mobile invertebrates was significantly greater in the seeded crevices than the unseeded crevices, followed by the seeded ridges, with the unseeded ridges containing the least (Fig 5iic, S5 and S6 Tables). Across all time points, the MaxN of the cryptobenthic fish was much higher adjacent to the seeded than the unseeded microhabitats, irrespective of whether they were crevices or ridges (Fig 5iid, Table 2, S5 Table).

### Effects of habitat structure and seeding with native oysters on the environmental variables

**Fig 6:**
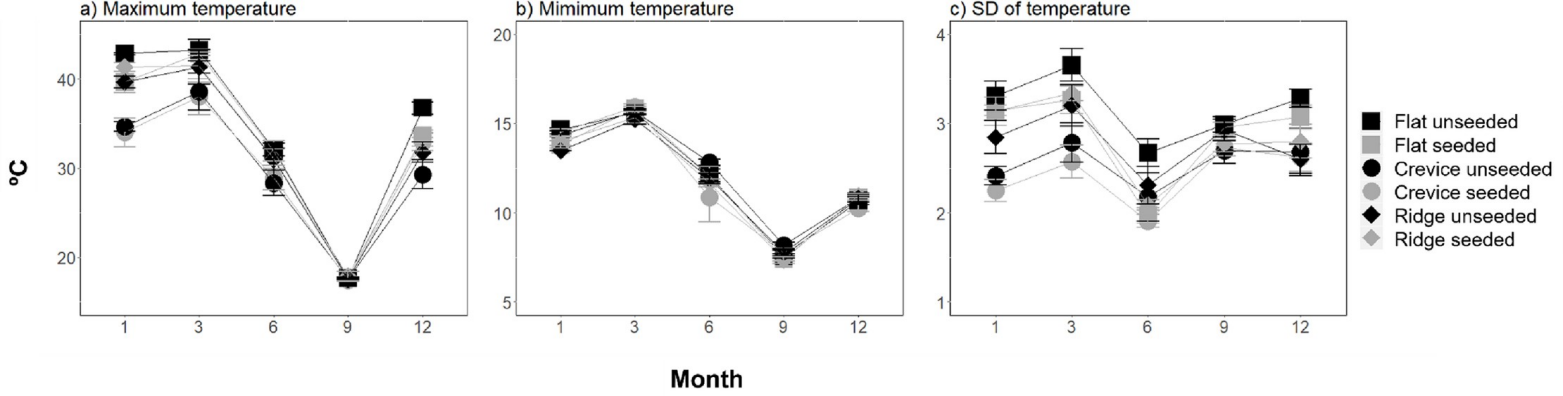
Effect of microhabitat type (flat vs crevice vs ridge) and seeding with native oysters (unseeded vs seeded) on the mean (+/-SE) (a) maximum, b) minimum and (b) standard deviation (SD) of temperature through time (months) since tile deployment (n = 5). Data are pooled across sites.

**Fig 7:**
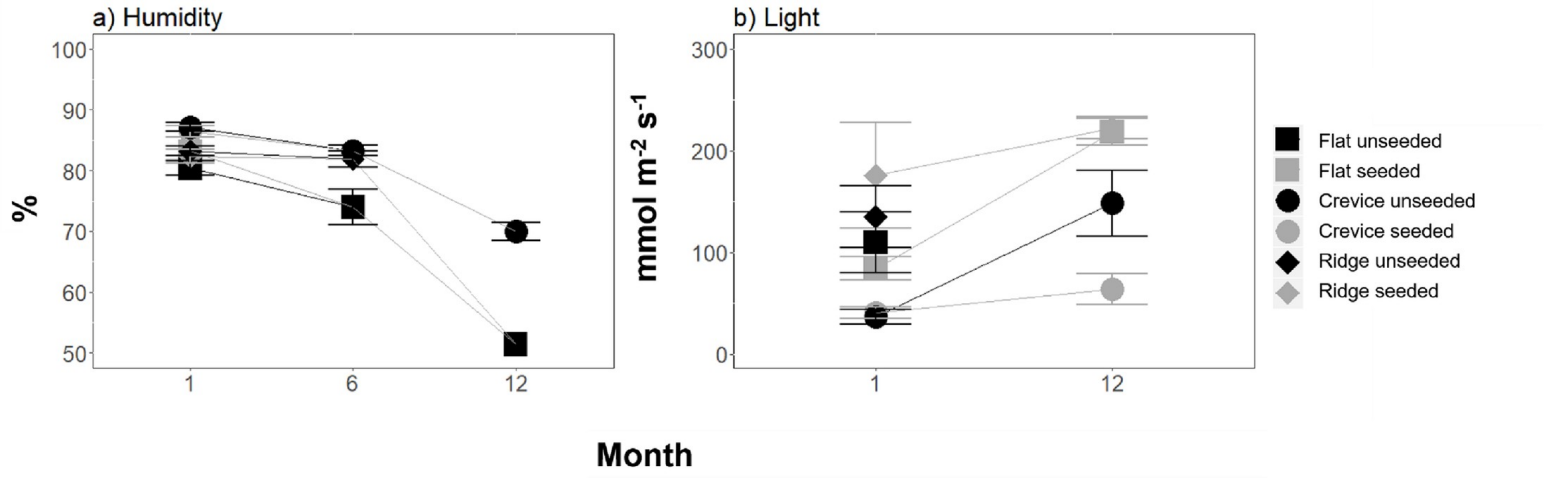
Effect of microhabitat (flat vs crevice vs ridge) and seeding (unseeded vs seeded) on the mean (+/-SE) (a) humidity (%) and (b) light (mmol m^-2^ s^-1^), through time (months), (n = 5). Data are pooled across sites.

Of the five environmental variables measured (maximum, minimum and standard deviation temperature, humidity and light), all but minimum temperature displayed significant treatment effects (Figs 6 and 7, S7 Table). Maximum and standard deviation temperature as well as light displayed effects of microhabitat that were independent of seeding, but which varied through time (Figs 6 and 7, S7 Table). At all-time points (excluding 9 months), the maximum temperature was significantly lower in the crevices than the flat tiles, but there were no detectable differences between the ridges and the flat tiles (Fig 6A, S7 Table). At 1, 3 and 12 months, but not 6 and 9 months, the crevices displayed a significantly smaller standard deviation of temperature as compared to flat tiles, but ridges displayed no difference to the flat tiles (Fig 6C, S7 Table). At both time points (1, 12 months) there were significantly higher light readings on flat tiles and on ridges, which displayed similar readings to one another, than in crevices (Fig 7B, S7 Table). In contrast, humidity displayed effects of microhabitat that were dependent on seeding and on time (Fig 7A, S7 Table). At all the three time points a similar humidity was recorded on flat tiles as on ridges, irrespective of seeding (Fig 7A, S7 Table). The crevices and flat microhabitats displayed no difference in humidity at 1 month and displayed differences only when unseeded at 6 months, but by 12 months crevices were more humid than flat microhabitats, independent of seeding (Fig 7A, S7 Table).

## Discussion

Our study provides the first experimental test of how habitat structure (crevices/ridges) and seeding with habitat-forming species (oysters) interact to influence the species density and abundances of four functional groups of organisms (sessile and mobile taxa and cryptobenthic and pelagic fishes) on vertically orientated substrate, in a highly modified urban marine environment. The two types of experimental manipulations generally had positive effects on the species density and abundances. Nevertheless, whether the effects of habitat structure or seeding with oysters were independent or interactive varied among functional groups.

The addition of habitat structure had a positive influence on the survival of seeded *S*. *glomerata* at the site with the highest morality and, at both sites, on the percentage cover of seeded and colonising oysters. Consequently, by 12 months, we found a similarly high percentage cover of oysters on seeded and unseeded complex tiles, although in the absence of habitat structure, oyster cover was greater on seeded than unseeded tiles. The generally greater survival and colonisation of oysters on the complex tiles relative to the flat tiles, appeared to be due to the protective role of the crevices, which reduced oyster mortality. The crevices may have protected oysters against both predation by pelagic fishes, which were more abundant around the seeded than unseeded tiles during the first 6 months, and summer low tide temperature extremes, which can be lethal to juvenile oysters [7, 48]. Maximum temperatures recorded in the crevices were up to 6.1°C cooler than on the ridges or flat plates in the first few months of our study, which corresponded to the Austral summer. Irrespective of the mechanism, these results highlight the importance of habitat structure in providing a refuge for juvenile oysters against predatory and environmental stressors.

Interestingly, there were no long-term effects of seeding with *S*. *glomerata* on the percentage cover of oysters on the complex tiles. Each of our study sites contained low density populations of wild oysters (relative to nearby rocky shores) [43] and oysters recruited in significant numbers to crevices, swamping the effects of seeding. Hence, at sites with a supply of *S*. *glomerata* recruits, the benefits of seeding may for enhancing percentage cover of oysters, in the longer-term, be minimal. Instead, seeding efforts may be better directed at sites where there are dispersal barriers to oyster settlement (perhaps introduced by coastal structures), but where environmental conditions are otherwise suitable for their survivorship, growth and reproduction [49].

The functional groups of organisms that responded most strongly to the manipulations of habitat structure and seeding with oysters were those whose body size most closely matched the dimensions of the interstices formed by the interventions. That is, our experimental manipulations which created microhabitats at the scale of cms, were most effective for enhancing the species density and/or abundances of benthic algae and invertebrates and cryptobenthic fishes but not pelagic fishes on seawalls [38, 50–52]. After 12 months, the species density of sessile algae and invertebrates and the percentage cover of *S*. *glomerata* was greater on the tiles for which one or both types of interventions were manipulated relative to the flat unseeded tiles. In contrast, the species density and abundances (counts or MaxN) of mobile taxa and cryptobenthic fishes on the tiles, were only influenced, positively, by seeding with oysters. These results suggest that even through the habitat structure provided by the seeded *S*. *glomerata* varied through time due to morality and growth of the oysters, there may be lasting effects of the initial seeding on mobile taxa and cryptobenthic fishes.

The similar species density of sessile taxa recorded between tiles irrespective of whether they contained habitat structure, seeded oysters or both suggests there was some redundancy in the effects of the treatments, or, alternatively, because oyster cover did not differ between the three treatments, that oyster cover was the driver of species density. Oysters may influence species density by: enhancing the surface area for attachment and grazing; by providing microhabitats between their shells that are protected from extreme low-tide air temperatures, desiccation stress and/or predators; through their biological functions of filter feeding, which can reduce the abundance of competitively dominant spores, or create currents that facilitate settlement; and through biodeposition which provides organic material [53–55]. The species density of mobile invertebrates and cryptobenthic fishes, may have responded more strongly to the initial seeding treatment than sessile taxa as they can move onto tiles within hours of their deployment to graze and utilise the microhabitat. In contrast, the colonisation of sessile taxa is dependent on larval settlement and may take longer to establish. Yet despite the rapid response of mobile species density to seeding with oysters, their abundances first displayed effects of habitat structure at 6 months and seeding at 12 months, Differences in the species density of sessile taxa among flat tiles and those with habitat structure may reflect the settlement choices of the organisms for cooler, shaded, moist and potentially less wave exposed surfaces [56], greater post-settlement mortality on unprotected surfaces, or reduced competition for space where more hard substrate is available for settlement.

Our experimental manipulations of seeded oysters were predicted to influence the species density and MaxN of pelagic fishes by enhancing prey availability and altering their foraging behaviour. However, contrary to our expectations, there were no temporally persistent effects of seeding on the species density or abundances of pelagic fishes. Previous research suggests that the relatively short-lived effect of seeding on the MaxN of pelagic fish, which disappeared after 6 months, was related to changing patterns of foraging on the tiles [7]. The most abundant pelagic fishes at 1 and 6 months were the Yellowfin bream (*Acanthopagrus australis*) and the Fanbelly leather jacket (*Monacanthus chinensis*). Both of these species are common around artificial habitats [57, 58] and are known predators of the Sydney rock oyster, *S*. *glomerata* [59–61], including those growing on the unprotected surfaces of seawalls [7, 62]. The absence of an effect of seeding on pelagic fishes after 6 months may reflect the loss of the oyster prey resource due to growth of oysters out of size classes most vulnerable to predation [7], and/or the complete removal of unprotected individuals from exposed surfaces, leaving only those in hard-to-get protected microhabitats.

On seawalls, the distribution and abundances of organisms are often limited by abiotic and biotic stressors [15, 63–67]. There are increasing efforts to eco-engineer missing microhabitats on seawalls to enhance the biodiversity and ecosystems functioning of their communities (e.g. clearance rates, carbon sequestration or bioprotection), [23, 38, 42, 67–69]. Small-scale experiments such as that conducted here can be useful in identifying appropriate eco-engineering approaches before they are scaled up. Our study clearly demonstrates that crevices can enhance shading and moisture retention and reduced the high temperature extremes at low tides compared with ridges and flat tiles. They may also reduce fish predation on the colonising taxa, although this was not directly quantified in this study.

Overall the presence of crevices increased the range of environmental conditions present on complex tiles which in turn could lead to greater co-existence of sessile and mobile taxa through portioning of resources [6]. In demonstrating that crevices support increased species density of sessile taxa and species density and abundances of mobile taxa as compared to ridges, our results support previous suggestions that the addition of crevices to homogenous, flat surfaces such as seawalls might be a successful ecoengineering approach [19]. Nevertheless, to fully assess the benefits, we would also need to compare the associated community of the creviced tiles to those of featureless seawalls.

Our research suggests that in addition to cervices, seeding with oysters influences the biodiversity of the sessile taxa, mobile taxa and cryptobenthic fishes supported by artificial substrata [70, 71]. We demonstrated that the species density of sessile taxa benefited from adding either habitat structure or seeding with oysters, while the species density and/or abundances of mobile taxa and cryptobenthic and pelagic fishes were primarily influenced, positively, by seeding. As different groups of organisms responded most strongly to different types of intervention, eco-engineering projects aimed at maximising the biodiversity of multiple functional groups might benefit from the creation of a variety of different types of microhabitats on any given structure, that increase the breadth of niche space available to organisms [19]. Alternatively, projects that focus on enhancing individual species (e.g. large predators or protected species) may want to target particular interventions that have the greatest benefit for these [72, 73].

Our small-scale tile experiment tested the effects of adding habitat structure and seeding with *S*. *glomerata* on the species density and abundances of four functional groups, at two sites and through time. Using this design, it was not possible to disentangle the effects of the initial treatment conditions from the effects of later colonising biota on the responses of the four functional groups. Further research, which manipulates different size classes and numbers of live and dead oysters, across multiple sites and locations, over which there is a greater range of species, is needed to inform the design of eco-engineering interventions. Such research should consider how site characteristics, the local species pool, and the scale of complexity relative to the size of the organisms influences outcomes of habitat manipulation [74]. When coupled with information on the costs and benefits of eco-engineering interventions, and the scales of interventions that maximise ecological benefits, the results of such studies will enable managers to make informed decisions regarding the best measures, given goals and site characteristics.

## Supporting information

S1 Table List of functional groups and species utilising each of the four experimental treatments during the experiment. Species/morphospecies are marked as present (+) or absent (-) based on sampling at month 12, whereby sessile and mobile species were censused using destructive sampling and pelagic and cryptic fish were sampled using GoPros *in situ*. If species were recorded at months 1 and/or 6, but not at month 12, they are listed but marked as absent at 12 months.
(DOCX)

S2 Table Results of generalised linear models testing the effects of habitat structure (flat vs. complex tiles) and microhabitat identity (crevice vs. ridge, nested within the complex tiles), on the number of live seeded *S*. *glomerata* sampled *in-situ*. The surface area of the tiles or microhabitats (offset), site and month (repeated measure) were also included in the model. Post hoc tests for significant factors of interest are shown. Tests significant at α = 0.05 are shown in bold.
(DOCX)

S3 Table Results of generalised linear models testing the effects of habitat structure (flat vs. complex) and microhabitat identity (crevice vs. ridge, nested within the complex tiles) and seeding with oysters (unseeded [US] vs. seeded [S]) on the percentage cover of *S*. *glomerata* sampled *in-situ* through time. The surface area of the tiles or microhabitats (offset), site and month (repeated measure) were also included in the model. Post hoc tests for significant factors of interest are shown. Tests significant at α = 0.05 are shown in bold.
(DOCX)

S4 Table Results of generalised linear models testing the effects of seeding with oysters (unseeded vs. seeded) and habitat structure (flat vs. complex tiles) or microhabitat identity (crevice vs. ridge, nested within complex tiles) on the species density sampled destructively at month 12 for the sessile algae and invertebrates (sessile) and mobile invertebrates (mobile) and *in-situ* at month 12 for cryptobenthic fishes and pelagic fishes. The surface area of tiles or microhabitats (offset) and site were also included in the model. Post hoc tests for significant factors of interest are shown. Tests significant at α = 0.05 are shown in bold.
(DOCX)

S5 Table Results of generalised linear models testing the effects of seeding with oysters (unseeded vs. seeded) and habitat structure (flat vs. complex tiles) or microhabitat identity (crevice vs. ridge, nested within complex tiles) on the percentage cover of sessile algae and invertebrates (sessile), the total abundance of mobile invertebrates (mobile) and the MaxN of each of cryptobenthic fishes and pelagic fishes. Functional groups were sampled *in-situ*, 1 6- and 12-months following deployment of tiles. The surface area of tiles or microhabitats (offset), site and month (repeated measure) were also included in the model. Post hoc tests for significant factors of interest are shown. Tests significant at α = 0.05 are shown in bold.
(DOCX)

S6 Table Results of generalised linear models testing the effects of seeding with oysters (unseeded vs. seeded) and habitat structure (flat vs. complex tiles) or microhabitat identity (crevice vs. ridge, nested within complex tiles) on the percentage cover of the sessile algae and invertebrates (sessile) and the total abundance of mobile invertebrates (mobile) sampled destructively at month 12. The surface area of tiles or microhabitats (offset), site and month (repeated measure) were also included in the model. Post hoc tests for significant factors of interest are shown. Tests significant at α = 0.05 are shown in bold.
(DOCX)

S7 Table Results of generalised linear models testing the effects of seeding with oysters (unseeded vs. seeded) and microhabitat identity (crevice or ridge each tested separately vs. flat) on the environmental variables (maximum temperature, minimum temperature, standard deviation temperature, average humidity and average light). Site and month (repeated measure) were also included in the model. Post hoc tests for significant factors of interest are shown. Tests significant at α = 0.05 are shown in bold.
(DOCX)
