# Supplementary material for: Interacting effects of habitat structure and seeding with oysters on the intertidal biodiversity of seawalls
Source: PLoS One. 2020 Jul 16;15(7):e0230807. doi: 10.1371/journal.pone.0230807 (PMC7365354; doi:10.1371/journal.pone.0230807)
Supplement: S1 Table — Species/morphospecies are marked as present (+) or absent (-) based on sampling at month 12, whereby sessile and mobile species were censused using destructive sampling and pelagic and cryptic fish were sampled using GoPros in situ. If species were recorded at months 1 and/or 6, but not at month 12, they are listed but marked as absent at 12 months. (DOCX) [file pone.0230807.s001.docx]

**Table S1:** List of functional groups and colonising species (excluding *S. glomerata* the seeded species) utilising each of the four experimental treatments throughout the experiment with both *in situ* and destructive sampling. Species/morphospecies are marked as present (+) or absent (-) based on sampling at month 12, whereby sessile and mobile species were censused using destructive sampling and pelagic and cryptic fish were sampled using GoPros *in situ*. If species were recorded at months 1 and/or 6, but not at month 12, they are listed but marked as absent across all treatments.

| **Functional group** | **Species** | **Reference** | Flat unseeded | Flat seeded | Complex unseeded | Complex seeded |
| --- | --- | --- | --- | --- | --- | --- |
| Pelagic fish | *Acanthopagrus australis* | (14) | - | + | - | + |
|  | *Achoerodus viridis* | (14) | - | - | - | - |
|  | *Brachaluteres jacksonianus* | (14) | - | - | - | - |
|  | *Cheilodactylus vestitus* | (14) | - | - | - | - |
|  | *Dicotylichthys punctulatus* | (14) | - | - | - | - |
|  | *Girella elevata* | (14) | - | - | - | - |
|  | *Girella tricuspidata* | (14) | - | - | - | - |
|  | *Meuschenia freycineti* | (14) | - | - | - | - |
|  | *Monacanthus chinensis* | (14) | - | - | - | - |
|  | *Neoodax balteatus* |  | - | - | - | + |
|  | *Prionurus microlepidotus* | (14) | - | - | - | - |
|  | *Pseudolabrus guentheri* | (14) | - | - | - | - |
|  | *Scobinichthys granulatus* | (14) | - | - | - | - |
| Cryptobenthic fish | *Bathygobius cocosensis* | (32) | - | - | + | + |
|  | *Omobranchus anolius* | (32) | + | + | + | + |
|  | *Omobranchus rotundiceps* | (32) | + | + | + | + |
|  | *Trinorfolkia clarkei* | (32) | + | - | - | + |
|  | *Parablennius intermedius* | (32) | + | + | + | + |
|  | *Pandaka lidwilli* | (32) | - | - | - | - |
|  | *Redigobius macrostoma* | (32) | - | + | - | + |
| Mobile invertebrates | **Polychaetes** |  |  |  |  |  |
|  | Polynoidae sp. | (49) | - | + | - | - |
|  | Phyllodocidae sp. |  | - | + | - | - |
|  | Nereidid sp. | (49) | + | + | - | + |
|  | Spionid sp. |  | - | + | - | + |
|  | Syllid sp. | (49) | - | + | - | + |
|  | **Sipunculida** | (49) | - | - | - | + |
|  | **Gastropod** |  |  |  |  |  |
|  | *Austrocochlea porcata* | (49) | - | + | + | + |
|  | *Austrocochlea concamerata* | (49) | - | - | - | + |
|  | *Afrolittorina acutispira* | (49) | - | - | - | - |
|  | *Bedeva paivae* |  | - | + | + | + |
|  | *Bembicium auratum* |  | + | + | + | + |
|  | *Bembicium nanum* | (49) | - | + | - | - |
|  | *Cellana tramoserica* | (49) | - | - | - | + |
|  | Gastropod: Spiral Unknown |  | - | - | - | + |
|  | *Littorina unifasciata* | (49) | + | + | + | + |
|  | Patelloida*latistrigata* | (49) | - | + | - | - |
|  | *Patelloida mimula* | (49) | + | + | + | + |
|  | *Patelloida mufria* | (49) | - | - | + | - |
|  | *Tenguella marginalba* |  | - | + | + | + |
|  | *Nerita atamentosa* | (49) | - | - | - | - |
|  | *Notoacmea flammea* | (49) | - | - | - | + |
|  | *Notoacmea* sp. | (49) | - | + | + | + |
|  | *Siphonaria denticulata* | (49) | + | + | + | + |
|  | *Siphonaria funiculata* | (49) | - | + | - | - |
|  | **Polyplacophora** |  |  |  |  |  |
|  | *Onithochiton quercinus* |  | - | + | + | + |
|  | *Sypharochiton pelliserpentis* |  | + | + | + | + |
|  | **Malacostraca** |  |  |  |  |  |
|  | *Ampithoe* sp. | (49) | + | + | + | + |
|  | *Cirolana harfordi* | (49) | + | + | + | + |
|  | *Corophium* sp. | (49) | - | - | + | + |
|  | Isopod 1 | (49) | + | + | + | + |
|  | Isopod 2 | (49) | - | - | - | - |
|  | *Parasesarma*erythrodactyla |  | + | + | + | + |
|  | *Pachygrapsus laevimanus* | (49) | - | + | + | - |
|  | **Bivalvia** |  |  |  |  |  |
|  | *Lasaea australis* | (49) | + | + | + | + |
|  | *Mytilus galloprovincialis* | (49) | + | + | + | + |
|  | *Tapes* sp. | (49) | - | + | + | - |
|  | *Trichomya hirsuta* | (49) | + | + | + | + |
|  | *Xenostrobus pulex* | (49) | - | - | - | + |
|  | **Collembola** | (49) | - | + | - | - |
| Sessile algae | **Chlorophyta** |  |  |  |  |  |
|  | Green mat | (49) | + | + | + | + |
|  | *Cladophora* sp. | (49) | - | - | + | + |
|  | *Ulva prolifera* | (49) | + | + | + | + |
|  | *Ulva lactuca* | (49) | - | - | - | + |
|  | ***Rhodophyta*** |  |  |  |  |  |
|  | Red turf | (49) | - | - | + | + |
|  | *Gelidium pusillum* | (49) | - | + | + | + |
|  | *Corallina officinalis* | (49) | - | + | + | + |
|  | **Phaeophyta** |  |  |  |  |  |
|  | *Ralfsia verrucosa* | (49) | + | + | + | + |
|  | Species 1 |  | - | + | - | + |
|  | Species 2 |  | - | + | - | - |
|  | Species 3 |  | - | + | + | + |
| Sessile invertebrates | **Cirripedia** |  |  |  |  |  |
|  | *Amphiblanus amphitrite* |  | - | + | - | - |
|  | *Hexaminius* sp. | (49) | + | + | + | + |
|  | *Elminius modestus* | (49) | + | + | + | + |
|  | **Ectoprota** |  |  |  |  |  |
|  | *Cryptosula pallasiana* | (49) | - | + | + | + |
|  | *Watersipora subtorquata* | (49) | - | - | + | - |
|  | **Polychaetes** |  |  |  |  |  |
|  | Spirorbidae sp. | (49) | - | + | + | + |
|  | **Porifera** |  |  |  |  |  |
|  | Porifera sp. | (49) | - | + | - | + |
