# Supplementary material for: Interacting effects of habitat structure and seeding with oysters on the intertidal biodiversity of seawalls
Source: PLoS One. 2020 Jul 16;15(7):e0230807. doi: 10.1371/journal.pone.0230807 (PMC7365354; doi:10.1371/journal.pone.0230807)
Supplement: S2 Table — The surface area of the tiles or microhabitats (offset), site and month (repeated measure) were also included in the model. Post hoc tests for significant factors of interest are shown. Tests significant at α = 0.05 are shown in bold. (DOCX) [file pone.0230807.s002.docx]

**Table S2:** Results of generalised linear models testing the effects of habitat structure (flat vs. complex tiles) or microhabitat identity (crevice vs. ridge, nested within the complex tiles) and month (repeated measure), on the number of live seeded *S. glomerata* sampled *in-situ.* The surface area of the tiles or microhabitats (offset) and site were also included in the model. Post hoc tests for significant factors of interest are shown. Tests significant at α = 0.05 are shown in bold.

| **Effects of adding habitats on the number of live seeded *S. glomerata*** | | | | | | | | |
| --- | --- | --- | --- | --- | --- | --- | --- | --- |
| **Factor** | **Value** | **Standard error** | **Z-value** | **P-value** | **Post hoc test** | **Estimate** | **Z ratio** | **P-value** |
| Habitat | -0.043 | 0.064 | -0.670 | 0.503 | Site 1, Month 1: Flat vs. Complex | 0.118 | 1.311 | 0.190 |
| Month | -0.029 | 0.007 | -4.000 | **<0.001** | Site 2, Month 1: Flat vs. Complex | -0.523 | -4.131 | **<0.001** |
| Habitat x Month | -0.002 | 0.011 | -0.145 | 0.885 | Site 1, Month 6: Flat vs. Complex | 0.223 | 0.234 | 0.194 |
|  |  | **Standard deviation** |  | **P-value** | Site 2, Month 6: Flat vs. Complex | -0.654 | -4.556 | **<0.001** |
| Site |  | 0.071 |  | **<0.001** | Site 1, Month 12: Flat vs. Complex | 0.074 | 0.696 | 0.486 |
| Habitat x Site |  | 0.097 |  | 0.063 | Site 2, Month 12: Flat vs. Complex | -1.065 | -5.935 | **<0.001** |
| Month x Site |  | 0.016 |  | **<0.001** |  |  |  |  |
| Habitat x Month x Site |  | 0.019 |  | **<0.001** |  |  |  |  |
| **Effects of adding microhabitats on the number of live seeded *S. glomerata*** | | | | | | | | |
| **Factor** | **Value** | **Standard error** | **Z-value** | **P-value** | **Post hoc test** | **Estimate** | **Z ratio** | **P-value** |
| Microhabitat | 0.113 | 0.128 | 0.878 | 0.380 | Site 1, Month 1: Crevice vs. Ridge | -0.088 | -0.687 | 0.492 |
| Month | -1.113 | 0.121 | -1.140 | **<0.001** | Site 2, Month 1: Crevice vs. Ridge | 0.764 | 4.739 | **<0.001** |
| Microhabitat x Month | -0.207 | 0.018 | -1.213 | 0.862 | Site 1, Month 6: Crevice vs. Ridge | 0.002 | 0.012 | 0.991 |
|  |  | **Standard deviation** |  | **P-value** | Site 2, Month 6: Crevice vs. Ridge | 1.245 | 6.382 | **<0.001** |
| Site |  | 0.127 |  | 0.862 | Site 1, Month 12: Crevice vs. Ridge | 0.140 | 1.022 | 0.307 |
| Microhabitat x Site |  | 0.212 |  | **<0.001** | Site 2, Month 12 Crevice vs. Ridge | 1.241 | 6.733 | **<0.001** |
| Month x Site |  | 0.017 |  | 0.911 |  |  |  |  |
| Microhabitat x Month x Site |  | 0.234 |  | **<0.001** |  |  |  |  |
