# Supplementary material for: Interacting effects of habitat structure and seeding with oysters on the intertidal biodiversity of seawalls
Source: PLoS One. 2020 Jul 16;15(7):e0230807. doi: 10.1371/journal.pone.0230807 (PMC7365354; doi:10.1371/journal.pone.0230807)
Supplement: S3 Table — The surface area of the tiles or microhabitats (offset), site and month (repeated measure) were also included in the model. Post hoc tests for significant factors of interest are shown. Tests significant at α = 0.05 are shown in bold. (DOCX) [file pone.0230807.s003.docx]

**Table S3:** Results of generalised linear models testing the effects of habitat structure (flat vs. complex) and microhabitat identity (crevice vs. ridge, nested within the complex tiles) and seeding with oysters (unseeded [US] vs. seeded [S]) and month (repeated measure) on the percentage cover of *S. glomerata* sampled *in-situ* through time. The surface area of the tiles or microhabitats (offset) and site were also included in the model. Post hoc tests for significant factors of interest are shown. Tests significant at α = 0.05 are shown in bold.

| **Effects of adding habitats and seeding with oysters on the percentage cover of *S. glomerata*** | | | | | | | | |
| --- | --- | --- | --- | --- | --- | --- | --- | --- |
| **Factor** | **Value** | **Standard error** | **Z-value** | **P-value** | **Post hoc tests** | **Estimate** | **Z ratio** | **P-value** |
| Habitat | 1.906 | 0.318 | 5.985 | **<0.001** | Month 1:  Flat US vs Flat S | -1.163 | -3.211 | **0.019** |
| Seeding | 1.914 | 0.323 | 5.917 | **<0.001** | Month 1 Flat US vs. Complex US | -1.025 | -2.808 | 0.066 |
| Month | 0.235 | 0.0272 | 8.642 | **<0.001** | Month 1: Flat US vs. Complex S | -1.089 | -3.008 | **0.036** |
| Habitat x Seeding | -0.054 | 0.029 | -1.834 | 0.067 | Month 1: Flat S vs. Complex US | 1.137 | 1.543 | **0.017** |
| Habitat x Month | -2.202 | 0.377 | -5.836 | **<0.001** | Month 1: Flat S vs. Complex S | 0.073 | 0.294 | 0.999 |
| Seeding x Month | -0.118 | 0.031 | -3.846 | **0.001** | Month 1: Complex US vs. Complex S | -1.074 | -2.008 | **0.029** |
| Habitat x Seeding x Month | 1.028 | 0.018 | 1.502 | **<0.001** | Month 6:  Flat US vs Flat S | -0.383 | -4.496 | **0.001** |
|  |  | **Standard deviation** |  | **P-value** | Month 6: Flat US vs. Complex US | 0.027 | 0.359 | 0.999 |
| Site |  | 1.385 |  | 0.179 | Month 6: Flat US vs. Complex S | -0.356 | -4.224 | **0.001** |
| Habitat x Site |  | 1.559 |  | 0.300 | Month 6: Flat S vs. Complex US | 0.169 | 2.330 | **0.021** |
| Seeding x Site |  | 1.594 |  | 0.256 | Month 6: Flat S vs. Complex S | 0.028 | 0.359 | 0.999 |
| Site x Month |  | 0.129 |  | 0.606 | Month 6: Complex US vs. Complex S | -0.493 | -4.740 | **0.001** |
| Habitat x Seeding x Site |  | 1.925 |  | 0.296 | Month 12: Flat US vs Flat S | -0.283 | -3.970 | **0.001** |
| Habitat x Seeding x Site x Month |  | 0.185 |  | 0.636 | Month 12: Flat US vs. Complex US | -0.667 | -10.221 | **<0.001** |
|  |  |  |  |  | Month 12: Flat US vs. Complex S | -0.525 | -7.677 | **<0.001** |
|  |  |  |  |  | Month 12: Flat S vs. Complex US | 0.038 | 1.458 | 0.111 |
|  |  |  |  |  | Month 12: Flat S vs. Complex S | -0.032 | -1.773 | 0.260 |
|  |  |  |  |  | Month 12: Complex US vs. Complex S | 0.153 | 2.802 | >0.066 |
| **Effects of adding microhabitats on percentage cover of *S. glomerata*** | | | | | | | | |
| **Factor** | **Value** | **Standard error** | **Z-value** | **P-value** | **Post hoc test** | **Estimate** | **Z ratio** | **P-value** |
| Microhabitat | 0.798 | 0.695 | 1.494 | 0.135 | Month 1:  Crevice S vs Crevice US | 3.104 | 9.600 | **<0.001** |
| Seeding | 5.138 | 0.513 | 10.026 | **<0.001** | Month 1 Crevice S vs. Ridge S | 0.171 | 1.742 | 0.549 |
| Month | 0.479 | 0.044 | 11.136 | **<0.001** | Month 1: Crevice S vs. Ridge US | 3.132 | 9.685 | **<0.001** |
| Microhabitat x Seeding | -1.290 | 0.707 | -1.825 | 0.068 | Month 1: Crevice US vs. Ridge S | -2.892 | -8.895 | **<0.001** |
| Microhabitat x Month | -0.219 | 0.062 | -3.580 | **0.001** | Month 1: Crevice US vs. Ridge US | 0.028 | 0.061 | 1.000 |
| Seeding x Month | -0.427 | 0.044 | -9.676 | **<0.001** | Month 1: Ridge S vs. Ridge US | 2.919 | 8.979 | **<0.001** |
| Microhabitat x Seeding x Month | 0.232 | 0.063 | 3.692 | **0.001** | Month 6:  Crevice S vs Crevice US | 3.492 | 10.877 | **<0.001** |
|  |  | **Standard deviation** |  | **P-value** | Month 6: Crevice S vs. Ridge S | 0.686 | 7.254 | **<0.001** |
| Site |  | 0.718 |  | 0.622 | Month 6: Crevice S vs. Ridge US | 3.520 | 10.962 | **<0.001** |
| Site x Month |  | 0.061 |  | 0.281 | Month 6: Crevice US vs. Ridge S | -2.806 | -8.623 | **<0.001** |
| Microhabitats x Site |  | 0.994 |  | 0.882 | Month 6: Crevice US vs. Ridge US | 0.028 | 0.061 | 1.000 |
| Seeding x Site |  | 0.729 |  | 0.879 | Month 6: Ridge S vs. Ridge US | 2.833 | 8.707 | **<0.001** |
| Microhabitat x Month x Site |  | 0.086 |  | 0.621 | Month 12:  Crevice S vs Crevice US | -0.017 | -0.271 | 0.999 |
| Seeding x Month x Site |  | 0.062 |  | 0.858 | Month 12: Crevice S vs. Ridge S | 0.473 | 6.725 | **<0.001** |
| Microhabitat x Seeding x Site |  | 1.012 |  | 0.978 | Month 12: Crevice S vs. Ridge US | 1.373 | 13.974 | **<0.001** |
| Microhabitat x Seeding x Site x Month |  | 0.890 |  | 0.255 | Month 12: Crevice US vs. Ridge S | 0.490 | 6.977 | **<0.001** |
|  |  |  |  |  | Month 12: Crevice US vs. Ridge US | 1.390 | 14.159 | **<0.001** |
|  |  |  |  |  | Month 12: Ridge S vs. Ridge US | 0.900 | 8.665 | **<0.001** |
