# Supplementary material for: Interacting effects of habitat structure and seeding with oysters on the intertidal biodiversity of seawalls
Source: PLoS One. 2020 Jul 16;15(7):e0230807. doi: 10.1371/journal.pone.0230807 (PMC7365354; doi:10.1371/journal.pone.0230807)
Supplement: S4 Table — The surface area of tiles or microhabitats (offset) and site were also included in the model. Post hoc tests for significant factors of interest are shown. Tests significant at α = 0.05 are shown in bold. (DOCX) [file pone.0230807.s004.docx]

**Table S4:** Results of generalised linear models testing the effects of habitat structure (flat vs. complex tiles) or microhabitat identity (crevice vs. ridge, nested within complex tiles) and seeding with oysters (unseeded [US] vs. seeded [S]) on the species density sampled destructively at month 12 for the sessile algae and invertebrates (sessile) and mobile invertebrates (mobile) and *in-situ* at month 12 for cryptobenthic fishes and pelagic fishes. The surface area of tiles or microhabitats (offset) and site were also included in the model. Post hoc tests for significant factors of interest are shown. Tests significant at α = 0.05 are shown in bold.

| **Effects of habitat structure and seeding on the species density of sessile taxa** | | | | | | | | |
| --- | --- | --- | --- | --- | --- | --- | --- | --- |
| **Factor** | **Value** | **Standard error** | **Z-value** | **P-value** | **Post hoc test** | **Estimate** | **Z-ratio** | **P-value** |
| Habitat | 0.864 | 0.324 | 2.665 | **0.005** | Flat US vs. Complex US | -0.899 | -2.822 | **0.035** |
| Seeding | 0.864 | 0.324 | 2.665 | **0.008** | Flat US vs. Flat S | -0.875 | -2.666 | **0.039** |
| Habitat x Seeding | -0.766 | 0.353 | -2.168 | **0.030** | Flat US vs. Complex S | -0.998 | -2.891 | **0.020** |
|  |  | **Standard deviation** |  | **P-value** | Complex US vs. Flat S | 0.600 | 0.887 | 0.812 |
| Site |  | 0.001 |  | 0.999 | Complex US vs. Complex S | -0.500 | -0.739 | 0.881 |
| Habitat x Site |  | 0.011 |  | 0.558 | Flat S vs. Complex S | -1.100 | -1.626 | 0.379 |
| Seeding x Site |  | 0.109 |  | 0.770 |  |  |  |  |
| Habitat x Seeding x Site |  | 0.007 |  | 0.605 |  |  |  |  |
| **Effects of habitat structure and seeding on the species density of mobile invertebrates** | | | | | | | | |
| **Factor** | **Value** | **Standard error** | **Z-value** | **P-value** | **Post hoc test** | **Estimate** | **Z-ratio** | **P-value** |
| Habitat | 0.263 | 0.298 | 0.882 | 0.367 | US vs. S | -0.706 | -5.876 | **<0.001** |
| Seeding | 1.131 | 0.275 | 4.400 | **<0.001** |  |  |  |  |
| Habitat x Seeding | -0.438 | 0.352 | -1.245 | 0.094 |  |  |  |  |
|  |  | **Standard deviation** |  | **P-value** |  |  |  |  |
| Site |  | 0.001 |  | 0.762 |  |  |  |  |
| Habitat x Site |  | 0.009 |  | 0.094 |  |  |  |  |
| Seeding x Site |  | 0.166 |  | 0.394 |  |  |  |  |
| Habitat x Seeding x Site |  | 0.134 |  | 0.261 |  |  |  |  |
| **Effects of habitat structure and seeding on the species density cryptobenthic fishes** | | | | | | | | |
| **Factor** | **Value** | **Standard error** | **Z-value** | **P-value** | **Post hoc test** | **Estimate** | **Z-ratio** | **P-value** |
| Habitat | -0.102 | 0.357 | -0.285 | 0.302 | US vs. S | -0.490 | -2.215 | **0.027** |
| Seeding | 0.525 | 0.236 | 2.228 | **0.026** |  |  |  |  |
| Habitat x Seeding | 0.097 | 0.443 | 0.219 | 0.274 |  |  |  |  |
|  |  | **Standard deviation** |  | **P-value** |  |  |  |  |
| Site |  | 0.001 |  | 0.302 |  |  |  |  |
| Habitat x Site |  | 0.001 |  | 0.463 |  |  |  |  |
| Seeding x Site |  | 0.001 |  | 0.464 |  |  |  |  |
| Habitat x Seeding x Site |  | 0.054 |  | 0.603 |  |  |  |  |
| **Effects of habitat structure and seeding on the species density pelagic fishes** | | | | | | | | |
| **Factor** | **Value** | **Standard error** | **Z-value** | **P-value** | **Post hoc test** | **Estimate** | **Z-ratio** | **P-value** |
| Habitat | -0.074 | 0.474 | -0.155 | 1.000 | NA |  |  |  |
| Seeding | 0.095 | 0.437 | 0.218 | 1.000 |  |  |  |  |
| Habitat x Seeding | 0.087 | 0.632 | 0.138 | 0.420 |  |  |  |  |
|  |  | **Standard deviation** |  | **P-value** |  |  |  |  |
| Site |  | 0.001 |  | 1.000 |  |  |  |  |
| Habitat x Site |  | 0.001 |  | 1.000 |  |  |  |  |
| Seeding x Site |  | 0.001 |  | 0.420 |  |  |  |  |
| Habitat x Seeding x Site |  | 0.0601 |  | 0.568 |  |  |  |  |
| **Effects of microhabitats and seeding on the species density of sessile taxa** | | | | | | | | |
| **Factor** | **Value** | **Standard error** | **Z-value** | **P-value** | **Post hoc test** | **Estimate** | **Z-ratio** | **P-value** |
| Microhabitat | -0.517 | 0.206 | -2.512 | **0.012** | Crevice vs. Ridge | 0.427 | 2.990 | **0.003** |
| Seeding | -0.033 | 0.181 | -0.182 | 0.882 |  |  |  |  |
| Microhabitat x Seeding | 0.178 | 0.286 | 0.628 | 0.579 |  |  |  |  |
|  |  | **Standard deviation** |  | **P-value** |  |  |  |  |
| Site |  | 0.001 |  | 1.000 |  |  |  |  |
| Microhabitat x Site |  | 0.011 |  | 0.181 |  |  |  |  |
| Seeding x Site |  | 0.109 |  | 0.484 |  |  |  |  |
| Microhabitat x Seeding x Site |  | 0.148 |  | 0.580 |  |  |  |  |
| **Effects of microhabitats and seeding on the species density of mobile invertebrates** | | | | | | | | |
| **Factor** | **Value** | **Standard error** | **Z-value** | **P-value** | **Post hoc test** | **Estimate** | **Z-ratio** | **P-value** |
| Microhabitat | -1.266 | 0.254 | -4.984 | **<0.001** | Crevice US vs. Ridge US | 4.776 | 9.567 | **<0.001** |
| Seeding | 0.288 | 0.160 | 1.806 | 0.071 | Crevice US vs. Crevice S | 0.112 | 0.427 | 0.271 |
| Microhabitat x Seeding | 0.794 | 0.304 | 2.614 | **0.009** | Crevice US vs. Ridge S | 0.550 | 2.084 | 0.841 |
|  |  | **Standard deviation** |  | **P-value** | Ridge US vs. Crevice S | -4.664 | -8.234 | **<0.001** |
| Site |  | 0.009 |  | 0.994 | Ridge US vs, Ridge S | -4.226 | -7.456 | **<0.001** |
| Microhabitat x Site |  | 0.001 |  | 1.000 | Crevice S vs. Ridge S | 0.438 | 8.315 | **<0.001** |
| Seeding x Site |  | 0.001 |  | 0.355 |  |  |  |  |
| Microhabitat x Seeding x Site |  | 0.001 |  | 1.000 |  |  |  |  |
| **Effects of microhabitats and seeding on the species density of cryptobenthic fishes** | | | | | | | | |
| **Factor** | **Value** | **Standard error** | **Z-value** | **P-value** | **Post hoc test** | **Estimate** | **Z-ratio** | **P-value** |
| Microhabitat | 0.241 | 0.369 | 0.654 | 0.545 | US vs. S | -0.493 | -2.157 | **0.031** |
| Seeding | 0.576 | 0.278 | 1.717 | **0.040** |  |  |  |  |
| Microhabitat x Seeding | -0.086 | 0.458 | -0.188 | 0.976 |  |  |  |  |
|  |  | **Standard deviation** |  | **P-value** |  |  |  |  |
| Site |  | 0.001 |  | 1.000 |  |  |  |  |
| Microhabitat x Site |  | 0.001 |  | 1.000 |  |  |  |  |
| Seeding x Site |  | 0.001 |  | 1.000 |  |  |  |  |
| Microhabitat x Seeding x Site |  | 0.001 |  | 1.000 |  |  |  |  |
