# Supplementary material for: Interacting effects of habitat structure and seeding with oysters on the intertidal biodiversity of seawalls
Source: PLoS One. 2020 Jul 16;15(7):e0230807. doi: 10.1371/journal.pone.0230807 (PMC7365354; doi:10.1371/journal.pone.0230807)
Supplement: S5 Table — Functional groups were sampled in-situ, 1 6- and 12-months following deployment of tiles. The surface area of tiles or microhabitats (offset), site and month (repeated measure) were also included in the model. Post hoc tests for significant factors of interest are shown. Tests significant at α = 0.05 are shown in bold. (DOCX) [file pone.0230807.s005.docx]

**Table S5:** Results of generalised linear models testing the effects of habitat structure (flat vs. complex tiles) or microhabitat identity (crevice vs. ridge, nested within complex tiles), seeding with oysters (unseeded [US] vs. seeded [S]) and month (repeated measure) on the percentage cover of sessile algae and invertebrates (sessile), the total abundance of mobile invertebrates (mobile) and the MaxN of each of cryptobenthic fishes and pelagic fishes. Functional groups were sampled *in-situ*, 1 6- and 12-months following deployment of tiles. The surface area of tiles or microhabitats (offset), and site were also included in the model. Post hoc tests for significant factors of interest are shown. Tests significant at α = 0.05 are shown in bold.

| **Effects of adding habitat structure and seeding on the percentage cover of sessile taxa** | | | | | | | | |
| --- | --- | --- | --- | --- | --- | --- | --- | --- |
| **Factor** | **Value** | **Standard error** | **T-value** | **P-value** | **Post hoc test** | **Estimate** | **Z ratio** | **P-value** |
| Habitat | 2.008 | 1.416 | 1.418 | 0.159 | NA |  |  |  |
| Seeding | 1.647 | 1.484 | 1.109 | 0.269 |  |  |  |  |
| Month | 0.234 | 0.121 | 1.928 | 0.057 |  |  |  |  |
| Habitat x Seeding | -2.314 | 1.754 | -1.320 | 0.189 |  |  |  |  |
| Habitat x Month | -0.081 | 0.133 | -0.614 | 0.540 |  |  |  |  |
| Seeding x Month | -0.128 | 0.142 | -0.904 | 0.368 |  |  |  |  |
| Habitat x Seeding x Month | 0.050 | 0.153 | 0.332 | 0.740 |  |  |  |  |
|  |  | **Standard deviation** |  | **P-value** |  |  |  |  |
| Site |  | 0.143 |  | 0.183 |  |  |  |  |
| Habitat x Site |  | 1.601 |  | 0.334 |  |  |  |  |
| Seeding x Site |  | 1.705 |  | 0.314 |  |  |  |  |
| Site x Month |  | 0.134 |  | 0.565 |  |  |  |  |
| Habitat x Site x Month |  | 0.153 |  | 0.740 |  |  |  |  |
| Habitat x Seeding x Site |  | 2.078 |  | 0.346 |  |  |  |  |
| Seeding x Site x Month |  | 0.165 |  | 0.514 |  |  |  |  |
| Habitat x Seeding x Site x Month |  | 0.204 |  | 0.692 |  |  |  |  |
| **Effects of habitat structure and seeding on the abundance of mobile invertebrates** | | | | | | | | |
| **Factor** | **Value** | **Standard error** | **Z-value** | **P-value** | **Post hoc test** |  |  |  |
| Habitat | 1.103 | 0.537 | 2.054 | **0.041** | Month 1, Site 1:  Flat US vs. Complex US | -0.139 | -0.299 | 0.991 |
| Seeding | 2.505 | 0.486 | 5.159 | **<0.001** | Month 1, Site 1: Flat US vs. Flat S | 0.001 | 0.001 | 1.000 |
| Month | 0.295 | 0.041 | 7.241 | **<0.001** | Month 1, Site 1: Flat US vs. Complex S | -0.220 | -0.474 | 0.965 |
| Habitat x Seeding | -0.830 | 0.582 | -1.428 | 0.154 | Month 1, Site 1: Complex US vs. Flat S | 0.139 | 0.299 | 0.998 |
| Habitat x Month | -0.049 | 0.049 | -1.014 | 0.311 | Month 1, Site 1: Complex US vs. Complex S | -0.081 | -0.175 | 0.998 |
| Seeding x Month | -0.163 | 0.045 | -3.674 | **0.001** | Month 1, Site 1: Flat S vs. Complex S | -0.220 | -0.474 | 0.965 |
| Habitat x Seeding x Month | 1.091 | 1.054 | 2.695 | **0.026** | Month 1, Site 2:  Flat US vs. Complex US | 0.074 | 0.001 | 1.000 |
|  |  | Standard deviation |  | P-value | Month 1, Site 2: Flat US vs. Flat S | -17.792 | -0.007 | >0.05 |
| Site |  | -1.409 |  | **<0.001** | Month 1, Site 2: Flat US vs. Complex S | -16.620 | -0.006 | >0.05 |
| Habitat x Site |  | 1.060 |  | **<0.001** | Month 1, Site 2: Complex US vs. Flat S | -17.866 | -0.007 | >0.05 |
| Seeding x Site |  | 0.960 |  | **<0.001** | Month 1, Site 2: Complex US vs. Complex S | -16.694 | -0.007 | >0.05 |
| Site x Month |  | 0.071 |  | **0.036** | Month 1, Site 2: Flat US vs. Complex S | 1.173 | 1.015 | >0.05 |
| Habitat x Site x Month |  | 0.009 |  | 0.739 | Month 6, Site 1:  Flat US vs. Complex US | 0.002 | 0.005 | 1.000 |
| Habitat x Seeding x Site |  | 0.126 |  | 0.692 | Month 6, Site 1: Flat US vs. Flat S | -1.849 | -3.970 | **0.002** |
| Seeding x Site x Month |  | 1.257 |  | 0.692 | Month 6, Site 1: Flat US vs. Complex S | -1.916 | -4.138 | **0.001** |
| Habitat x Seeding x Site x Month |  | 1.344 |  | **<0.001** | Month 6, Site 1: Complex US vs. Flat S | -1.841 | -3.974 | **0.002** |
|  |  |  |  |  | Month 6, Site 1: Complex US vs. Complex S | -1.918 | -4.142 | **<0.001** |
|  |  |  |  |  | Month 6, Site 1: Flat S vs. Complex S | -0.078 | -0.168 | 0.999 |
|  |  |  |  |  | Month 6, Site 2:  Flat US vs. Complex US | -0.045 | -0.091 | 0.995 |
|  |  |  |  |  | Month 6 Site 2: Flat US vs. Flat S | -1.118 | -2.424 | **0.040** |
|  |  |  |  |  | Month 6, Site 2: Flat US vs. Complex S | -1.738 | -3.736 | **<0.001** |
|  |  |  |  |  | Month 6, Site 2: Complex US vs. Flat S | -1.074 | -1.156 | **0.035** |
|  |  |  |  |  | Month 6, Site 2: Complex US vs. Complex S | -1.693 | -1.698 | **0.010** |
|  |  |  |  |  | Month 6, Site 2: Flat S vs. Complex S | -0.620 | -1.518 | 0.325 |
|  |  |  |  |  | Month 12, Site 1: Flat US vs. Complex US | -0.434 | -0.936 | 0.786 |
|  |  |  |  |  | Month 12, Site 1: Flat US vs. Flat S | -0.479 | -1.035 | 0.730 |
|  |  |  |  |  | Month 12, Site 1: Flat US vs. Complex S | -1.326 | -2.862 | **0.031** |
|  |  |  |  |  | Month 12, Site 1: Complex US vs. Flat S | -0.046 | -0.100 | 0.999 |
|  |  |  |  |  | Month 12, Site 1: Complex US vs. Complex S | -2.892 | -6.150 | **<0.001** |
|  |  |  |  |  | Month 12, Site 1: Flat S vs. Complex S | -3.790 | -6.349 | **<0.001** |
|  |  |  |  |  | Month 12, Site 2:  Flat US vs. Complex US | -0.141 | -1.659 | 0.999 |
|  |  |  |  |  | Month 12, Site 2: Flat US vs. Flat S | -0.416 | -2.544 | 0.056 |
|  |  |  |  |  | Month 12, Site 2: Flat US vs. Complex S | -0.465 | -3.580 | **0.002** |
|  |  |  |  |  | Month 12, Site 2: Complex US vs. Flat S | 0.333 | 2.025 | 0.065 |
|  |  |  |  |  | Month 12, Site 2: Complex US vs. Complex S | -0.549 | -4.214 | **0.001** |
|  |  |  |  |  | Month 12, Site 2: Flat S vs. Complex S | -0.881 | -5.893 | **<0.001** |
| **Effects of habitat structure and seeding on the MaxN of cryptobenthic fishes** | | | | | | | | |
| **Factor** | **Value** | **Standard error** | **T-value** | **P-value** | **Post hoc test** | **Estimate** | **Z ratio** | **P-value** |
| Habitat | 0.135 | 0.274 | 0.494 | 0.624 | US vs, S | -0.265 | -3.205 | **0.002** |
| Seeding | 0.604 | 0.276 | 2.193 | **0.031** |  |  |  |  |
| Month | 0.053 | 0.025 | 2.133 | **0.046** |  |  |  |  |
| Habitat x Seeding | -0.082 | 0.388 | -0.211 | 0.834 |  |  |  |  |
| Habitat x Month | -0.011 | 0.038 | -0.300 | 0.765 |  |  |  |  |
| Seeding x Month | -0.035 | 0.034 | -1.011 | 0.314 |  |  |  |  |
| Habitat x Seeding x Month | 0.002 | 0.052 | 0.049 | 0.961 |  |  |  |  |
|  |  | **Standard deviation** |  | **P-value** |  |  |  |  |
| Site |  | 0.283 |  | 0.659 |  |  |  |  |
| Habitat x Site |  | 0.402 |  | 0.430 |  |  |  |  |
| Seeding x Site |  | 0.397 |  | 0.101 |  |  |  |  |
| Site x Month |  | 0.038 |  | 0.765 |  |  |  |  |
| Habitat x Site x Month |  | 0.053 |  | 0.961 |  |  |  |  |
| Habitat x Seeding x Site |  | 0.569 |  | 0.196 |  |  |  |  |
| Seeding x Site x Month |  | 0.050 |  | 0.529 |  |  |  |  |
| Habitat x Seeding x Site x Month |  | 0.073 |  | 0.667 |  |  |  |  |
| **Effects of habitat structure and seeding on the MaxN of pelagic fishes** | | | | | | | | |
| **Factor** | **Value** | **Standard error** | **T-value** | **P-value** | **Post hoc test** | **Estimate** | **Z ratio** | **P-value** |
| Habitat | 0.022 | 0.046 | 0.485 | 0.629 | Month 1, US vs. S | -0.197 | -6.828 | **<0.001** |
| Seeding | 0.120 | 0.045 | 2.652 | **0.009** | Month 6, US vs. S | -0.028 | -1.046 | 0.321 |
| Month | -0.004 | 0.004 | -1.006 | 0.316 | Month 12, US vs. S | -0.030 | -1.146 | 0.302 |
| Habitat x Seeding | -0.091 | 0.065 | -1.395 | 0.166 |  |  |  |  |
| Habitat x Month | -0.002 | 0.006 | -0.396 | 0.692 |  |  |  |  |
| Seeding x Month | -0.059 | 0.057 | -2.028 | **0.030** |  |  |  |  |
| Habitat x Seeding x Month | 0.010 | 0.008 | 1.286 | 0.201 |  |  |  |  |
|  |  | **Standard deviation** |  | **P-value** |  |  |  |  |
| Site |  | 0.064 |  | 0.280 |  |  |  |  |
| Habitat x Site |  | 0.092 |  | 0.733 |  |  |  |  |
| Seeding x Site |  | 0.090 |  | 0.372 |  |  |  |  |
| Site x Month |  | 0.008 |  | 0.319 |  |  |  |  |
| Habitat x Site x Month |  | 0.011 |  | 0.796 |  |  |  |  |
| Habitat x Seeding x Site |  | 0.130 |  | 0.067 |  |  |  |  |
| Seeding x Site x Month |  | 0.012 |  | 0.796 |  |  |  |  |
| Habitat x Seeding x Site x Month |  | 0.017 |  | 0.241 |  |  |  |  |
| **Effects of microhabitats and seeding on the percentage cover of sessile algae and invertebrates** | | | | | | | | |
| **Factor** | **Value** | **Standard error** | **T-value** | **P-value** | **Post hoc test** | **Estimate** | **Z ratio** | **P-value** |
| Microhabitat | -0.174 | 0.881 | -0.198 | 0.844 | NA |  |  |  |
| Seeding | -0.628 | 1.027 | -0.612 | 0.542 |  |  |  |  |
| Month | 0.154 | 0.060 | 2.587 | **0.011** |  |  |  |  |
| Microhabitat x Seeding | 0.900 | 1.369 | 0.657 | 0.512 |  |  |  |  |
| Microhabitat x Month | -0.008 | 0.088 | -0.094 | 0.925 |  |  |  |  |
| Seeding x Month | -0.017 | 0.104 | -0.171 | 0.864 |  |  |  |  |
| Microhabitat x Seeding x Month | -0.059 | 0.142 | -0.422 | 0.673 |  |  |  |  |
|  |  | **Standard deviation** |  | **P-value** |  |  |  |  |
| Site |  | 0.738 |  | 0.293 |  |  |  |  |
| Microhabitats x Site |  | 1.112 |  | 0.783 |  |  |  |  |
| Seeding x Site |  | 1.236 |  | 0.840 |  |  |  |  |
| Month x Site |  | 0.075 |  | 0.463 |  |  |  |  |
| Microhabitat x Month x Site |  | 0.113 |  | 0.693 |  |  |  |  |
| Seeding x Month x Site |  | 0.127 |  | 0.913 |  |  |  |  |
| Microhabitat x Seeding x Site |  | 1.677 |  | 0.940 |  |  |  |  |
| Microhabitat x Seeding x Site x Month |  | 0.175 |  | 0.794 |  |  |  |  |
| **Effects of microhabitats and seeding on the abundances of mobile invertebrates** | | | | | | | | |
| **Factor** | **Value** | **Standard error** | **T-value** | **P-value** | **Post hoc test** | **Estimate** | **Z ratio** | **P-value** |
| Microhabitat | -2.680 | 0.886 | -3.025 | **0.002** | Month 1, Crevice US vs. Ridge US | 0.210 | 0.490 | 0.998 |
| Seeding | 1.060 | 0.246 | 4.295 | **<0.001** | Month 1, Crevice US vs. Crevice S | 0.001 | 0.001 | 1.000 |
| Month | 0.175 | 0.020 | 8.627 | **<0.001** | Month 1, Crevice US vs. Ridge S | -0.252 | -0.659 | 0.992 |
| Microhabitat x Seeding | -0.179 | 1.003 | -0.179 | 0.858 | Month 1, Ridge US vs. Crevice S | -0.210 | -0.490 | 0.998 |
| Microhabitat x Month | -0.060 | 0.092 | -0.653 | 0.513 | Month 1, Ridge US vs. Ridge S | -0.462 | -1.143 | 0.893 |
| Seeding x Month | -0.050 | 0.024 | -2.057 | **0.039** | Month 1, Crevice S vs. Ridge S | -0.252 | -0.659 | 0.992 |
| Microhabitat x Seeding x Month | 3.613 | 2.287 | 2.580 | **0.011** | Month 6, Crevice US vs. Ridge US | 1.774 | 6.148 | **<0.001** |
|  |  | **Standard deviation** |  | **P-value** | Month 6, Crevice US vs. Crevice S | -0.465 | -2.947 | 0.054 |
| Site |  | 0.447 |  | 0.237 | Month 6, Crevice US vs. Ridge S | 1.456 | 5.496 | **<0.001** |
| Microhabitats x Site |  | 2.117 |  | 0.593 | Month 6, Ridge US vs. Crevice S | -2.239 | -7.890 | **<0.001** |
| Seeding x Site |  | 0.605 |  | 0.291 | Month 6, Ridge US vs. Ridge S | -0.318 | -0.896 | 0.963 |
| Month x Site |  | 0.041 |  | 0.138 | Month 6, Crevice S vs. Ridge S | 1.921 | 7.396 | **<0.001** |
| Microhabitat x Month x Site |  | 0.189 |  | 0.254 | Month 12, Crevice US vs. Ridge US | 2.116 | 9.243 | **<0.001** |
| Seeding x Month x Site |  | 0.055 |  | 0.213 | Month 12, Crevice US vs. Crevice S | -0.486 | -5.147 | **<0.001** |
| Microhabitat x Seeding x Site |  | 0.206 |  | 0.197 | Month 12, Crevice US vs. Ridge S | 1.028 | 7.048 | **<0.001** |
| Microhabitat x Seeding x Site x Month |  | 0.209 |  | 0.244 | Month 12, Ridge US vs. Crevice S | -2.602 | -11.608 | **<0.001** |
|  |  |  |  |  | Month 12, Ridge US vs. Ridge S | -1.088 | -4.348 | **0.002** |
|  |  |  |  |  | Month 12, Crevice S vs. Ridge S | 1.514 | 10.953 | **<0.001** |
| **Effects of microhabitats and seeding on the MaxN of cryptobenthic fishes** | | | | | | | | |
| **Factor** | **Value** | **Standard error** | **T-value** | **P-value** | **Post hoc test** | **Estimate** | **Z ratio** | **P-value** |
| Microhabitat | -0.038 | 0.242 | -0.158 | 0.874 | US vs. S | -0.294 | -3.949 | **0.001** |
| Seeding | 0.616 | 0.242 | 2.536 | **0.012** |  |  |  |  |
| Month | 0.066 | 0.023 | 2.863 | **0.005** |  |  |  |  |
| Microhabitat x Seeding | -0.194 | 0.343 | -0.565 | 0.573 |  |  |  |  |
| Microhabitat x Month | -0.019 | 0.032 | -0.601 | 0.549 |  |  |  |  |
| Seeding x Month | -0.053 | 0.032 | -1.636 | 0.105 |  |  |  |  |
| Microhabitat x Seeding x Month | 0.021 | 0.046 | 0.457 | 0.648 |  |  |  |  |
|  |  | **Standard deviation** |  | **P-value** |  |  |  |  |
| Site |  | 0.254 |  | 0.476 |  |  |  |  |
| Microhabitats x Site |  | 0.351 |  | 0.592 |  |  |  |  |
| Seeding x Site |  | 0.362 |  | 0.424 |  |  |  |  |
| Month x Site |  | 0.033 |  | 0.713 |  |  |  |  |
| Microhabitat x Month x Site |  | 0.047 |  | 0.505 |  |  |  |  |
| Seeding x Month x Site |  | 0.047 |  | 0.249 |  |  |  |  |
| Microhabitat x Seeding x Site |  | 0.507 |  | 0.808 |  |  |  |  |
| Microhabitat x Seeding x Site x Month |  | 0.066 |  | 0.903 |  |  |  |  |
