# Supplementary material for: Interacting effects of habitat structure and seeding with oysters on the intertidal biodiversity of seawalls
Source: PLoS One. 2020 Jul 16;15(7):e0230807. doi: 10.1371/journal.pone.0230807 (PMC7365354; doi:10.1371/journal.pone.0230807)
Supplement: S6 Table — The surface area of tiles or microhabitats (offset), site and month (repeated measure) were also included in the model. Post hoc tests for significant factors of interest are shown. Tests significant at α = 0.05 are shown in bold. (DOCX) [file pone.0230807.s006.docx]

**Table S6:** Results of generalised linear models testing the effects of habitat structure (flat vs. complex tiles) or microhabitat identity (crevice vs. ridge, nested within complex tiles), seeding with oysters (unseeded [US] vs. seeded [S]) on the percentage cover of the sessile algae and invertebrates (sessile) and the total abundance of mobile invertebrates (mobile) sampled destructively at month 12. The surface area of tiles or microhabitats (offset), and site were also included in the model. Post hoc tests for significant factors of interest are shown. Tests significant at α = 0.05 are shown in bold.

| **Effects of habitat structure and seeding on the percentage cover of sessile taxa** | | | | | | | | |
| --- | --- | --- | --- | --- | --- | --- | --- | --- |
| **Factor** | **Value** | **Standard error** | **T-value** | **P-value** | **Post hoc test** | **Estimate** | **T- ratio** | **P-value** |
| Habitat | 2.268 | 1.346 | 1.685 | 0.087 | NA |  |  |  |
| Seeding | 1.915 | 1.206 | 1.588 | 0.130 |  |  |  |  |
| Habitat x Seeding | -2.355 | 1.476 | -1.596 | 0.174 |  |  |  |  |
|  |  | **Standard deviation** |  | **P-value** |  |  |  |  |
| Site |  | 0.300 |  | 0.152 |  |  |  |  |
| Habitat x Site |  | 0.241 |  | 0.189 |  |  |  |  |
| Seeding x Site |  | 0.449 |  | 0.299 |  |  |  |  |
| Habitat x Seeding x Site |  | 0.454 |  | 0.266 |  |  |  |  |
| **Effects of habitat structure and seeding on the abundance of mobile invertebrates** | | | | | | | | |
| **Factor** | **Value** | **Standard error** | **Z-value** | **P-value** | **Post hoc test** | **Estimate** | **Z ratio** | **P-value** |
| Habitat | 1.221 | 0.132 | 9.255 | **<0.001** | Flat US vs. Complex US | -1.220 | -9.255 | **<0.001** |
| Seeding | 2.102 | 0.176 | 11.924 | **<0.001** | Flat US vs. Flat S | -2.102 | -11.924 | **<0.001** |
| Habitat x Seeding | -1.389 | 0.134 | -10.347 | **<0.001** | Flat US vs. Complex S | -1.933 | -10.384 | **<0.001** |
|  |  |  |  |  | Complex US vs. Flat S | -0.882 | -9.756 | **<0.001** |
| Site |  | 0.001 |  | 0.063 | Complex US vs. Complex S | -0.713 | -7.157 | **<0.001** |
| Habitat x Site |  | 0.051 |  | 0.258 | Flat S vs. Complex S | 0.095 | 1.936 | 0.189 |
| Seeding x Site |  | 0.045 |  | 0.240 |  |  |  |  |
| Habitat x Seeding x Site |  | 0.062 |  | 0.379 |  |  |  |  |
| **Effects of microhabitats and seeding on the percentage cover of sessile taxa** | | | | | | | | |
| **Factor** | **Value** | **Standard error** | **T-value** | **P-value** | **Post hoc test** | **Estimate** | **T- ratio** | **P-value** |
| Microhabitat | 0.372 | 1.716 | 0.216 | 0.864 | NA |  |  |  |
| Seeding | 1.891 | 2.779 | 0.680 | 0.247 |  |  |  |  |
| Microhabitat x Seeding | -0.236 | 1.844 | -1.28 | 0.699 |  |  |  |  |
|  |  | **Standard deviation** |  | **P-value** |  |  |  |  |
| Site |  | 0.001 |  | 1.000 |  |  |  |  |
| Microhabitat x Site |  | 0.001 |  | 0.997 |  |  |  |  |
| Seeding x Site |  | 0.248 |  | 0.556 |  |  |  |  |
| Microhabitat Seeding x Site |  | 0.348 |  | 0.700 |  |  |  |  |
| **Effects of microhabitats and seeding on the abundance of mobile invertebrates** | | | | | | | | |
| **Factor** | **Value** | **Standard error** | **Z-value** | **P-value** | **Post hoc test** | **Estimate** | **Z- ratio** | **P-value** |
| Microhabitat | -0.282 | 0.026 | -10.874 | **<0.001** | Crevice US vs. Ridge US | 2.932 | 10.774 | **<0.001** |
| Seeding | -0.131 | 0.0216 | -6.065 | **<0.001** | Crevice US vs. Crevice S | -1.033 | -4.602 | **<0.001** |
| Microhabitat x Seeding | 0.236 | 0.027 | 8.934 | **<0.001** | Crevice US vs. Ridge S | -1.575 | -2.547 | **0.010** |
|  |  | **Standard deviation** |  | **P-value** | Ridge US vs. Crevice S | -3.966 | -11.784 | **<0.001** |
| Site |  | 0.218 |  | 0.069 | Ridge US vs. Ridge S | -3.507 | **-**10.398 | **<0.001** |
| Microhabitat x Site |  | 0.336 |  | 0.0652 | Crevice S vs. Ridge S | 0.458 | 9.006 | **<0.001** |
| Seeding x Site |  | 0.175 |  | 0.367 |  |  |  |  |
| Microhabitat x Seeding x Site |  | 0.351 |  | 0.173 |  |  |  |  |
