# Supplementary material for: Interacting effects of habitat structure and seeding with oysters on the intertidal biodiversity of seawalls
Source: PLoS One. 2020 Jul 16;15(7):e0230807. doi: 10.1371/journal.pone.0230807 (PMC7365354; doi:10.1371/journal.pone.0230807)
Supplement: S7 Table — Site and month (repeated measure) were also included in the model. Post hoc tests for significant factors of interest are shown. Tests significant at α = 0.05 are shown in bold. (DOCX) [file pone.0230807.s007.docx]

**Table S7:** Results of generalised linear models testing the effects of microhabitat identity (crevice or ridge each tested separately vs. flat), seeding with oysters (unseeded [US] vs. seeded [S]) and month (repeated measure) on the environmental variables (maximum temperature, minimum temperature, standard deviation temperature, average humidity and average light). Site and were also included in the model. Post hoc tests for significant factors of interest are shown. Tests significant at α = 0.05 are shown in bold.

| **Effect of microhabitat (crevice) and seeding with oysters on maximum temperature** | | | | | | | | |
| --- | --- | --- | --- | --- | --- | --- | --- | --- |
| **Factor** | **Value** | **Standard error** | **T-value** | **P-value** | **Post hoc test** | **Estimate** | **Z-ratio** | **P-value** |
| Crevice | -8.166 | 1.956 | -4.173 | **<0.001** | Month 1: Flat vs. Crevice | 6.917 | 4.955 | **<0.001** |
| Seeding | -3.166 | 1.956 | -1.618 | 0.109 | Month 3: Flat vs. Crevice | 5.510 | 3.792 | **0.001** |
| Month | 0.416 | 1.956 | -5.494 | **<0.001** | Month 6: Flat vs. Crevice | 3.375 | 2.564 | **0.011** |
| Crevice x Seeding | 0.363 | 0.358 | 1.015 | 0.397 | Month 9: Flat vs. Crevice | 0.135 | 0.101 | 0.919 |
| Crevice x Month | 0.223 | 0.501 | 2.445 | **0.018** | Month 12: Flat vs. Crevice | 4.104 | 3.118 | **0.002** |
| Seeding x Month | -0.004 | 0.498 | -0.007 | 0.811 |  |  |  |  |
| Crevice x Seeding x Month | 0.280 | 0.719 | 0.390 | 0.369 |  |  |  |  |
|  |  | **Standard deviation** |  | **P-value** |  |  |  |  |
| Site |  | 0.001 |  | 1.000 |  |  |  |  |
| Crevice x Site |  | 0.003 |  | 0.789 |  |  |  |  |
| Seeding x Site |  | 0.003 |  | 1.000 |  |  |  |  |
| Month x Site |  | 0.001 |  | 0.085 |  |  |  |  |
| Crevice x Seeding x Site |  | 0.005 |  | 1.000 |  |  |  |  |
| Crevice x Month x Site |  | 0.005 |  | 0.814 |  |  |  |  |
| Seeding x Month x Site |  | 0.005 |  | 0.571 |  |  |  |  |
| Crevice x Seeding x Site x Month |  | 0.007 |  | 0.888 |  |  |  |  |
| **Effect of microhabitat (ridge) and seeding with oysters on maximum temperature** | | | | | | | | |
| **Factor** | **Value** | **Standard error** | **T-value** | **P-value** | **Post hoc test** | **Estimate** | **Z-ratio** | **P-value** |
| Ridge | -1.080 | 5.341 | -0.202 | 0.841 | NA |  |  |  |
| Seeding | -2.666 | 5.341 | -0.449 | 0.619 |  |  |  |  |
| Month | -3.724 | 5.342 | -0.697 | 0.488 |  |  |  |  |
| Ridge x Seeding | 3.009 | 7.554 | 0.398 | 0.691 |  |  |  |  |
| Ridge x Month | 0.004 | 0.721 | 0.004 | 0.997 |  |  |  |  |
| Seeding x Month | 0.310 | 0.720 | 0.431 | 0.667 |  |  |  |  |
| Ridge x Seeding x Month | -0.307 | 1.023 | -0.300 | 0.765 |  |  |  |  |
|  |  | **Standard deviation** |  | **P-value** |  |  |  |  |
| Site |  | 0.453 |  | 0.487 |  |  |  |  |
| Ridge x Site |  | 7.553 |  | 0.860 |  |  |  |  |
| Seeding x Site |  | 7.553 |  | 0.728 |  |  |  |  |
| Site x Month |  | 0.726 |  | 0.294 |  |  |  |  |
| Ridge x Seeding x Site |  | 10.682 |  | 0.857 |  |  |  |  |
| Ridge x Month x Site |  | 1.026 |  | 0.845 |  |  |  |  |
| Seeding x Month x Site |  | 1.026 |  | 0.518 |  |  |  |  |
| Ridge x Seeding x Site x Month |  | 1.451 |  | 0.640 |  |  |  |  |
| **Effect of microhabitat (crevice) and seeding with oysters on minimum temperature** | | | | | | | | |
| **Factor** | **Value** | **Standard error** | **T-value** | **P-value** | **Post hoc test** | **Estimate** | **Z-ratio** | **P-value** |
| Crevice | 0.183 | 1.442 | 0.127 | 0.899 | NA |  |  |  |
| Seeding | -0.709 | 1.476 | -0.480 | 0.630 |  |  |  |  |
| Month | -0.625 | 0.139 | -4.479 | **<0.001** |  |  |  |  |
| Crevice x Seeding | -0.181 | 2.121 | -0.085 | 0.932 |  |  |  |  |
| Crevice x Month | 0.068 | 0.196 | 0.347 | 0.729 |  |  |  |  |
| Seeding x Month | 0.095 | 0.198 | 0.479 | 0.633 |  |  |  |  |
| Crevice x Seeding x Month | -0.138 | 0.286 | -0.485 | 0.629 |  |  |  |  |
|  |  | **Standard deviation** |  | **P-value** |  |  |  |  |
| Site |  | 0.224 |  | 0.880 |  |  |  |  |
| Crevice x Site |  | 2.129 |  | 0.700 |  |  |  |  |
| Seeding x Site |  | 2.0873 |  | 0.803 |  |  |  |  |
| Month x Site |  | 0.200 |  | 0.593 |  |  |  |  |
| Crevice x Seeding x Site |  | 3.061 |  | 0.579 |  |  |  |  |
| Crevice x Month x Site |  | 0.286 |  | 0.994 |  |  |  |  |
| Seeding x Month x Site |  | 0.283 |  | 0.728 |  |  |  |  |
| Crevice x Seeding x Site x Month |  | 0.409 |  | 0.969 |  |  |  |  |
| **Effect of microhabitat (ridge) and seeding with oysters on minimum temperature** | | | | | | | | |
| **Factor** | **Value** | **Standard error** | **T-value** | **P-value** | **Post hoc test** | **Estimate** | **Z-ratio** | **P-value** |
| Ridge | -1.198 | 1.278 | -0.870 | 0.387 | NA |  |  |  |
| Seeding | -0.709 | 1.378 | -0.514 | 0.608 |  |  |  |  |
| Month | -0.626 | 0.131 | -4.795 | **<0.001** |  |  |  |  |
| Ridge x Seeding | 0.853 | 1.949 | 0.437 | 0.663 |  |  |  |  |
| Ridge x Month | 0.150 | 0.186 | 0.807 | 0.421 |  |  |  |  |
| Ridge x Seeding x Month | -0.096 | 0.264 | -0.357 | 0.714 |  |  |  |  |
|  |  | **Standard deviation** |  | **P-value** |  |  |  |  |
| Site |  | 1.378 |  | 0.871 |  |  |  |  |
| Ridge x Site |  | 1.949 |  | 0.797 |  |  |  |  |
| Seeding x Site |  | 1.949 |  | 0.790 |  |  |  |  |
| Month x Site |  | 0.187 |  | 0.567 |  |  |  |  |
| Ridge x Month x Site |  | 0.265 |  | 0.711 |  |  |  |  |
| Seeding x Month x Site |  | 0.265 |  | 0.710 |  |  |  |  |
| Ridge x Seeding x Site |  | 2.757 |  | 0.913 |  |  |  |  |
| Ridge x Seeding x Site x Month |  | 0.374 |  | 0.946 |  |  |  |  |
| **Effect of microhabitat (crevice) and seeding with oysters on the standard deviation of temperature** | | | | | | | | |
| **Factor** | **Value** | **Standard error** | **T-value** | **P-value** | **Post hoc test** | **Estimate** | **Z-ratio** | **P-value** |
| Crevice | -0.919 | 0.277 | -3.316 | **0.001** | Month 1, Flat vs, Crevice | 0.903 | 4.448 | **<0.001** |
| Seeding | -0.374 | 0.273 | -1.370 | 0.173 | Month 3, Flat vs. Crevice | 0.801 | 4.041 | **0.001** |
| Month | -0.026 | 0.026 | -1.008 | 0.315 | Month 6, Flat vs. Crevice | 0.278 | 1.438 | 0.143 |
| Crevice x Seeding | 0.166 | 0.399 | 0.415 | 0.678 | Month 9, Flat vs. Crevice | 0.249 | 1.284 | 0.218 |
| Crevice x Month | 0.445 | 0.377 | 1.195 | **0.023** | Month 12, Flat vs. Crevice | 0.527 | 2.723 | **0.007** |
| Seeding x Month | 0.012 | 0.037 | 0.337 | 0.736 |  |  |  |  |
| Crevice x Seeding x Month | 0.001 | 0.053 | 0.028 | 0.977 |  |  |  |  |
|  |  | **Standard deviation** |  | **P-value** |  |  |  |  |
| Site |  | 0.394 |  | 0.898 |  |  |  |  |
| Crevice x Site |  | 0.569 |  | 0.331 |  |  |  |  |
| Seeding x Site |  | 0.557 |  | 0.511 |  |  |  |  |
| Month x Site |  | 0.053 |  | 0.990 |  |  |  |  |
| Crevice x Month x Site |  | 0.076 |  | 0.369 |  |  |  |  |
| Seeding x Month x Site |  | 0.075 |  | 0.675 |  |  |  |  |
| Crevice x Seeding x Site |  | 0.817 |  | 0.539 |  |  |  |  |
| Crevice x Seeding x Site x Month |  | 0.109 |  | 0.623 |  |  |  |  |
| **Effect of microhabitat (ridge) and seeding with oysters on the standard deviation of temperature** | | | | | | | | |
| **Factor** | **Value** | **Standard error** | **T-value** | **P-value** | **Post hoc test** | **Estimate** | **Z-ratio** | **P-value** |
| Ridge | -0.192 | 0.282 | -0.481 | 0.631 | NA |  |  |  |
| Seeding | -0.191 | 0.398 | -0.479 | 0.633 |  |  |  |  |
| Month | -0.026 | 0.038 | -0.690 | 0.492 |  |  |  |  |
| Ridge x Seeding | 0.129 | 0.564 | 0.229 | 0.820 |  |  |  |  |
| Ridge x Month | -0.019 | 0.054 | -0.372 | 0.710 |  |  |  |  |
| Seeding x Month | -0.004 | 0.054 | -0.67 | 0.947 |  |  |  |  |
| Ridge x Seeding x Month | 0.001 | 0.076 | 0.018 | 0.986 |  |  |  |  |
|  |  | **Standard deviation** |  | **P-value** |  |  |  |  |
| Site |  | 0.398 |  | 0.899 |  |  |  |  |
| Ridge x Site |  | 0.563 |  | 0.480 |  |  |  |  |
| Seeding x Site |  | 0.563 |  | 0.515 |  |  |  |  |
| Month x Site |  | 0.054 |  | 0.991 |  |  |  |  |
| Ridge x Seeding x Site |  | 0.796 |  | 0.329 |  |  |  |  |
| Ridge x Month x Site |  | 0.076 |  | 0.637 |  |  |  |  |
| Seeding x Month x Site |  | 0.076 |  | 0.678 |  |  |  |  |
| Ridge x Seeding x Site x Month |  | 0.109 |  | 0.597 |  |  |  |  |
| **Effect of microhabitat (crevice) and seeding with oysters on the average humidity** | | | | | | | | |
| **Factor** | **Value** | **Standard error** | **T-value** | **P-value** | **Post hoc test** | **Estimate** | **Z-ratio** | **P-value** |
| Crevice | 6.141 | 3.107 | 1.977 | 0.052 | Month 1, Flat US vs. Crevice US | -0.081 | -1.036 | 0.170 |
| Seeding | 5.175 | 3.107 | 1.666 | 0.099 | Month 1, Flat US vs. Flat S | -0.035 | -1.287 | 0.574 |
| Month | -2.816 | 2.744 | -10.260 | **<0.001** | Month 1, Flat US vs. Crevice S | -0.072 | -1.703 | 0.411 |
| Crevice x Seeding | -7.022 | 4.394 | -1.598 | 0.114 | Month 1, Crevice US vs, Flat S | 0.047 | 1.775 | 0.306 |
| Crevice x Month | 1.471 | 3.881 | 3.789 | **0.003** | Month 1, Crevice US vs, Crevice S | 0.009 | 0.333 | 0.988 |
| Seeding x Month | 1.111 | 3.881 | 0.286 | 0.776 | Month 1, Flat S vs. Crevice S | -0.038 | -1.416 | 0.493 |
| Crevice x Seeding x Month | 6.122 | 5.489 | 3.112 | **0.002** | Month 6, Flat US vs. Crevice US | -0.232 | -6.730 | **<0.001** |
|  |  | **Standard deviation** |  | **P-value** | Month 6, Flat US vs. Flat S | -0.215 | -6.238 | **<0.001** |
| Site |  | 3.881 |  | 1.000 | Month 6, Flat US vs. Crevice S | -0.232 | -6.730 | **<0.001** |
| Crevice x Site |  | 4.394 |  | 0.856 | Month 6, Crevice US vs, Flat S | 0.017 | 0.492 | 0.961 |
| Seeding x Site |  | 4.394 |  | 0.759 | Month 6, Crevice US vs, Crevice S | 0.001 | 0.001 | 1.000 |
| Month x Site |  | 3.881 |  | 1.000 | Month 6, Flat S vs. Crevice S | -0.012 | -0.492 | 0.961 |
| Crevice x Seeding x Site |  | 6.214 |  | 0.884 | Month 12, Flat US vs. Crevice US | -0.348 | -13.012 | **<0.001** |
| Crevice x Month x Site |  | 5.489 |  | 0.397 | Month 12, Flat US vs. Flat S | -0.072 | -2.699 | 0.414 |
| Seeding x Month x Site |  | 5.489 |  | 0.819 | Month 12, Flat US vs. Crevice S | -0.348 | -13.012 | **<0.001** |
| Crevice x Seeding x Site x Month |  | 7.762 |  | 0.913 | Month 12, Crevice US vs, Flat S | 0.276 | 10.313 | **<0.001** |
|  |  |  |  |  | Month 12, Crevice US vs, Crevice S | 0.001 | 0.001 | 1.000 |
|  |  |  |  |  | Month 12, Flat S vs. Crevice S | -0.276 | -10.313 | **<0.001** |
| **Effect of microhabitat (ridge) and seeding with oysters on the average humidity** | | | | | | | | |
| **Factor** | **Value** | **Standard error** | **T-value** | **P-value** | **Post hoc test** | **Estimate** | **Z-ratio** | **P-value** |
| Ridge | 5.149 | 4.259 | 1.209 | 0.230 | NA |  |  |  |
| Seeding | 1.961 | 4.259 | 0.460 | 0.646 |  |  |  |  |
| Month | -2.916 | 0.377 | -7.482 | **<0.001** |  |  |  |  |
| Ridge x Seeding | -3.921 | 6.025 | -0.651 | 0.517 |  |  |  |  |
| Ridge x Month | -0.231 | 0.533 | -0.433 | 0.666 |  |  |  |  |
| Seeding x Month | -0.182 | 0.532 | -0.344 | 0.732 |  |  |  |  |
| Ridge x Seeding x Month | 0.365 | 0.753 | 0.486 | 0.628 |  |  |  |  |
|  |  | **Standard deviation** |  | **P-value** |  |  |  |  |
| Site |  | 4.259 |  | 0.453 |  |  |  |  |
| Ridge x Site |  | 6.024 |  | 0.869 |  |  |  |  |
| Seeding x Site |  | 6.024 |  | 0.823 |  |  |  |  |
| Month x Site |  | 0.532 |  | 0.582 |  |  |  |  |
| Ridge x Seeding x Site |  | 8.629 |  | 0.797 |  |  |  |  |
| Ridge x Month x Site |  | 0.752 |  | 0.904 |  |  |  |  |
| Seeding x Month x Site |  | 0.752 |  | 0.867 |  |  |  |  |
| Ridge x Seeding x Site x Month |  | 1.072 |  | 0.847 |  |  |  |  |
| **Effect of microhabitat (crevice) and seeding with oysters on the average light** | | | | | | | | |
| **Factor** | **Value** | **Standard error** | **T-value** | **P-value** | **Post hoc test** | **Estimate** | **Z-ratio** | **P-value** |
| Crevice | -1.163 | 0.625 | -3.248 | **0.002** | Flat vs. Crevice | 1.230 | 6.989 | **<0.001** |
| Seeding | -0.109 | 0.625 | -0.174 | 0.863 |  |  |  |  |
| Month | 0.128 | 0.049 | 2.583 | **0.013** |  |  |  |  |
| Crevice x Seeding | -0.362 | 0.884 | -0.410 | 0.684 |  |  |  |  |
| Crevice x Month | 0.007 | 0.072 | 0.097 | 0.923 |  |  |  |  |
| Seeding x Month | -0.034 | 0.069 | -0.486 | 0.629 |  |  |  |  |
| Crevice x Seeding x Month | -0.008 | 0.101 | -0.078 | 0.938 |  |  |  |  |
|  |  | **Standard deviation** |  | **P-value** |  |  |  |  |
| Site |  | 0.879 |  | 0.451 |  |  |  |  |
| Crevice x Site |  | 1.244 |  | 0.590 |  |  |  |  |
| Seeding x Site |  | 1.244 |  | 0.946 |  |  |  |  |
| Month x Site |  | 0.098 |  | 0.573 |  |  |  |  |
| Crevice x Seeding x Site |  | 1.758 |  | 0.614 |  |  |  |  |
| Crevice x Month x Site |  | 0.143 |  | 0.644 |  |  |  |  |
| Seeding x Month x Site |  | 0.138 |  | 0.959 |  |  |  |  |
| Crevice x Seeding x Site x Month |  | 0.200 |  | 0.823 |  |  |  |  |
| **Effect of microhabitat (ridge) and seeding with oysters on the average light** | | | | | | | | |
| **Factor** | **Value** | **Standard error** | **T-value** | **P-value** | **Post hoc test** | **Estimate** | **Z-ratio** | **P-value** |
| Ridge | 0.502 | 0.351 | 1.430 | 0.159 | NA |  |  |  |
| Seeding | -0.066 | 0.351 | -0.188 | 0.851 |  |  |  |  |
| Month | 0.099 | 0.027 | 3.591 | **<0.001** |  |  |  |  |
| Ridge x Seeding | 0.237 | 0.497 | 0.476 | 0.635 |  |  |  |  |
| Ridge x Month | -0.041 | 0.039 | -1.069 | 0.289 |  |  |  |  |
| Seeding x Month | -0.037 | 0.039 | -0.954 | 0.344 |  |  |  |  |
| Ridge x Seeding x Month | -0.019 | 0.056 | -0.356 | 0.724 |  |  |  |  |
|  |  | **Standard deviation** |  | **P-value** |  |  |  |  |
| Site |  | 0.351 |  | 0.063 |  |  |  |  |
| Ridge x Site |  | 0.496 |  | 0.288 |  |  |  |  |
| Seeding x Site |  | 0.496 |  | 0.864 |  |  |  |  |
| Month x Site |  |  |  |  |  |  |  |  |
| Ridge x Seeding x Site |  | 0.702 |  | 0.908 |  |  |  |  |
| Ridge x Month x Site |  | 0.057 |  | 0.305 |  |  |  |  |
| Seeding x Month x Site |  | 0.055 |  | 0.898 |  |  |  |  |
| Ridge x Seeding x Site x Month |  | 0.080 |  | 0.827 |  |  |  |  |
